# Sterol biosensor reveals LAM-family Ltc1-dependent sterol flow to endosomes upon Arp2/3 inhibition

**DOI:** 10.1083/jcb.202001147

**Published:** 2020-04-22

**Authors:** Magdalena Marek, Vincent Vincenzetti, Sophie G. Martin

**Affiliations:** Department of Fundamental Microbiology, University of Lausanne, Switzerland

## Abstract

Sterols are crucial components of biological membranes, which are synthetized in the ER and accumulate in the plasma membrane (PM). Here, by applying a genetically encoded sterol biosensor (D4H), we visualize a sterol flow between PM and endosomes in the fission yeast *Schizosaccharomyces pombe*. Using time-lapse and correlative light-electron microscopy, we found that inhibition of Arp2/3-dependent F-actin assembly promotes the reversible relocalization of D4H from the PM to internal sterol-rich compartments (STRIC) labeled by synaptobrevin Syb1. Retrograde sterol internalization to STRIC is independent of endocytosis or an intact Golgi, but depends on Ltc1, a LAM/StARkin-family protein localized to ER-PM contact sites. The PM in *ltc1Δ* cells over-accumulates sterols and upon Arp2/3 inhibition forms extended ER-interacting invaginations, indicating that sterol transfer contributes to PM size homeostasis. Anterograde sterol movement from STRIC is independent of canonical vesicular trafficking but requires Arp2/3, suggesting a novel role for this complex. Thus, transfer routes orthogonal to vesicular trafficking govern the flow of sterols in the cell.

## Teaser

This work establishes the genetically encoded fluorescent sensor D4H to track sterol-rich membranes in yeast cells. Unexpectedly, disruption of the actin cytoskeleton leads to movement of sterols from the plasma membrane to endosomes. Internalization is independent of canonical vesicle trafficking and instead depends on the sterol transfer protein Ltc1.

## Introduction

Sterols are critical components of biological membranes with fundamental roles in cellular physiology. They confer essential biophysical properties to biomembranes, and regulate vesicular trafficking and signal transduction. Moreover, they serve as precursors of hormones, bile acids, vitamin D, and energy storage molecules. While sterols can be either synthesized in cells or taken up from the environment, dysfunctions in their trafficking or metabolism cause severe pathologies, such as Niemann–Pick disease type C, hypercholesterolemia, or sitosterolemia. In fungi, the sterol molecule ergosterol is essential for viability and serves as a major target of antifungal drugs.

Although sterols are synthesized in the ER, their concentration within the ER does not exceed 0.5–1 mol% of total lipids (Luo et al., 2017). Instead, it increases along the secretory pathway, reaching ∼40 mol% at the plasma membrane (PM; Maxfield and van Meer, 2010). Within the PM, sterols cluster with other lipids, especially sphingolipids, forming membrane subdomains that range from the nanoscale to several micrometers (Alvarez et al., 2007; Makushok et al., 2016; Sezgin et al., 2017).

How sterol domains are formed and maintained, and how sterols are transported between different organelles, have been the subject of intense investigation. Previous work in mammalian and yeast cells showed that the transport of newly synthetized cholesterol from the ER to the PM is fast, with a half-time of a few minutes (Baumann et al., 2005; Georgiev et al., 2011), and does not occur through the canonical secretory pathway. Indeed, collapse of the Golgi apparatus by Brefeldin A treatment in mammalian cells or secretory pathway block with temperature-sensitive mutants in yeast has no or little effect on sterol transport rates (Baumann et al., 2005; Heino et al., 2000; Urbani and Simoni, 1990). Instead, sterol molecules are thought to be carried between membranes by lipid transfer proteins (LTPs; Wong et al., 2017).

We can distinguish two major classes of proteins implicated in sterol transport (Luo et al., 2019). The first is the family of oxysterol-binding protein (OSBP)–related proteins (ORP) known in* Saccharomyces cerevisiae* as oxysterol-binding protein homologues (OSH). Not all proteins in this family transport sterol, but they bind phosphoinositides as common ligands, leading to the hypothesis that some ORPs (e.g., mammalian OSBP and *S. cerevisiae* Osh4/Kes1) transport sterol between membranes by counter-exchange with phosphoinositides (Antonny et al., 2018). Surprisingly, knockout of all seven OSH proteins in yeast did not significantly affect bidirectional sterol transport, suggesting that either OSH’s primary function is not bulk sterol transport, or that there exists a large degree of redundancy with other sterol transport pathways (Georgiev et al., 2011; Raychaudhuri et al., 2006).

The second class of sterol transport proteins is part of the large StARkin superfamily, which possesses a steroidogenic acute regulatory transfer (StART) or StART-like lipid-binding domain (Wong and Levine, 2016). The StART domain, able to bind sterols, phospho- and/or sphingolipids, defines the STARD family, which is absent from fungi and Archaea. The StART-like domain, also known as VASt (VAD1 analogue of StART; Khafif et al., 2014), binds cholesterol and ergosterol, and promotes the transfer of sterol molecules between membranes (Gatta et al., 2018; Horenkamp et al., 2018; Jentsch et al., 2018; Tong et al., 2018). In *S. cerevisiae*, members of this protein family, named lipid transfer proteins anchored at membrane contact sites (LAM), are typically enriched at ER-organelle contact sites (Elbaz-Alon et al., 2015; Gatta et al., 2015; Murley et al., 2015). Lam1-4 localize to ER-PM contact sites, with Lam2/Ysp2 involved in retrograde sterol transport (Gatta et al., 2015); Lam6/Ltc1 localizes to contact sites between ER, mitochondria, and vacuoles (Elbaz-Alon et al., 2015; Murley et al., 2015); Lam5/Ltc2 may localize to contact sites between the ER and the late Golgi (Weill et al., 2018). The mammalian homologues, called GramD1 or Aster, also localize to ER-PM contact sites and contribute to retrograde transport of exogenously provided cholesterol (Besprozvannaya et al., 2018; Sandhu et al., 2018). Intriguingly, while most LTPs localize to dedicated organelle contact sites, *S. cerevisiae* mutants lacking all six known ER-PM tethering proteins show normal anterograde and only partially diminished retrograde sterol transport (Quon et al., 2018).

Yeast cells, such as *S. cerevisiae* and* Schizosaccharomyces pombe*, have simple endomembrane systems. For instance, the trans-Golgi network (TGN) in *S. cerevisiae* also serves as an early and late endosomal compartment (Day et al., 2018). Both organisms harbor a single, well-characterized, endocytic pathway that uses Arp2/3-mediated actin assembly to generate forces for vesicle internalization from the PM to the TGN (Goode et al., 2015; Lacy et al., 2018). Anterograde vesicular trafficking from the TGN to the PM relies on transport along actin cables, assembled by the formin For3 in fission yeast, and tethering at the PM by the exocyst complex (Bendezú and Martin, 2011; Feierbach et al., 2004; TerBush et al., 1996; Wang et al., 2002). Our extended knowledge of protein trafficking contrasts sharply with that of sterol transport, which remains mostly unknown.

A major difficulty in studying intracellular sterol transport lies in its visualization. The fluorescent antibiotic filipin, a widely used polyene macrolide-based sterol dye, is not suitable for prolonged live imaging due to toxicity (Alvarez et al., 2007). Other approaches rely on application of fluorescently labeled sterol analogues, which, however, carry bulky chemical groups that can alter sterol behavior (Sezgin et al., 2016). Moreover, yeast cells only take them up in specific mutant backgrounds or growth conditions (Jacquier and Schneiter, 2012; Marek et al., 2014). Domain 4 (D4) of the bacterial toxin perfringolysin O, which binds membranes containing ≥30 mol% sterols and can be expressed in cells with no obvious toxicity, offers an interesting alternative (Shimada et al., 2002). Part of the reason for the high detection threshold is that perfringolysin O detects free sterol in the membrane, but not sterol in complex with sphingolipids (Das et al., 2014). An improved allele, called D4H, capable of binding membranes with 20 mol% sterols (Johnson et al., 2012; Maekawa and Fairn, 2015), represents a promising genetically encoded biosensor for tracking sterols in vivo (Maekawa et al., 2016).

Here, we applied the D4H probe to visualize sterol-rich membranes in the fission yeast *S. pombe* and discover a route of sterol movement from the PM, revealed by depolymerization of the actin cytoskeleton. We show that sterols undergo retrograde trafficking from the PM to endosomes, in a manner strictly dependent on a single, previously uncharacterized StARkin-protein Ltc1, and return to the PM by means independent of vesicular trafficking.

## Results

### D4H as a bioprobe for intracellular sterol visualization

**Figure S1.**
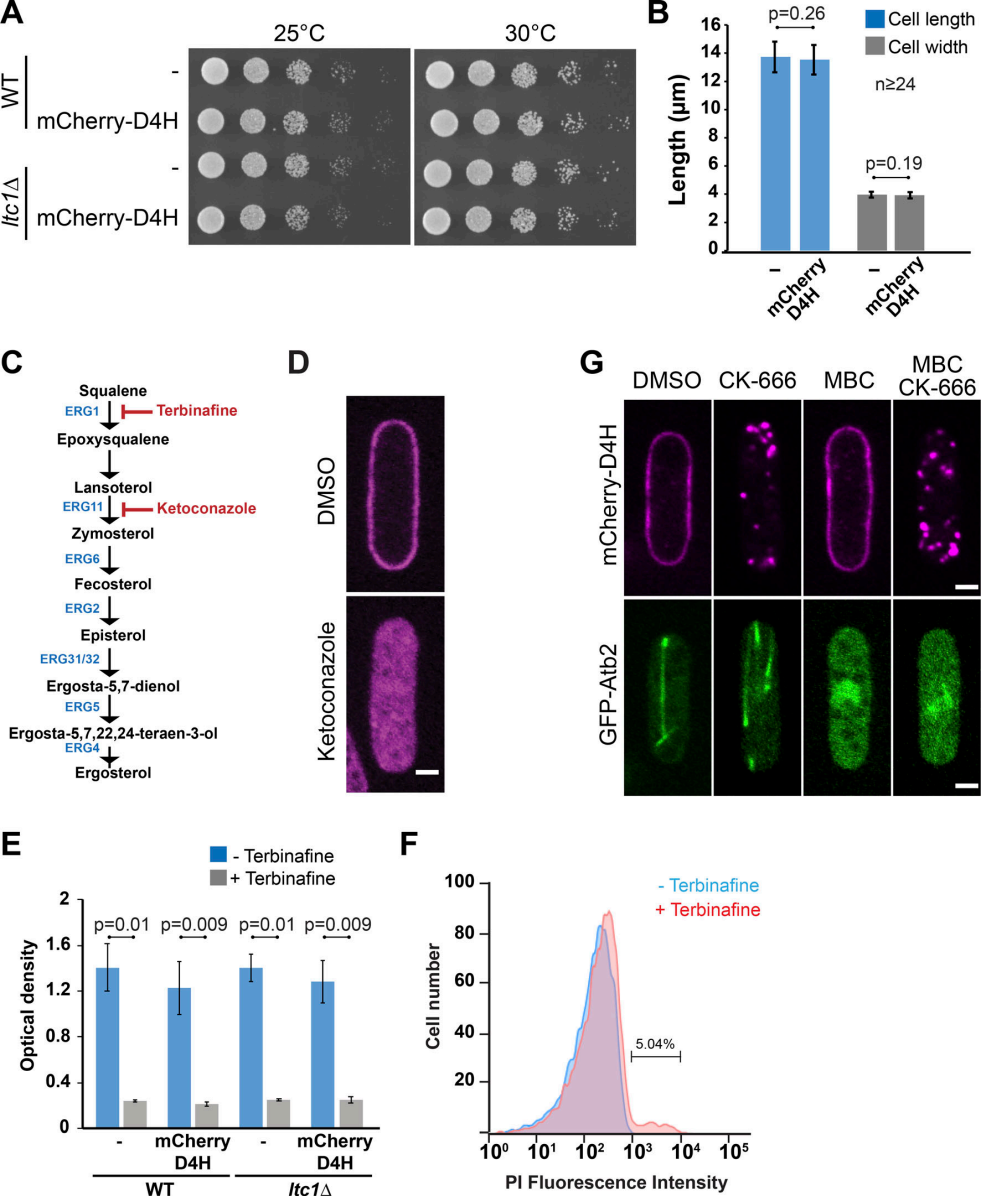
**Controls for lack of D4H toxicity, ketoconazole and terbinafine treatment, and microtubule depolymerization. (A)** Expression of mCherry-D4H and deletion of *ltc1* do not influence growth capability of *S. pombe* cells. Serial dilutions of WT and *ltc1Δ* mutant expressing or not mCherry-D4H were compared for ability to grow at 25°C and 30°C. **(B)** Expression of mCherry-D4H does not influence cell length and width. Results represent three independent experiments. **(C)** Ergosterol biosynthesis pathway. **(D)** Cells treated with 200 mM ketoconazole or solvent (DMSO) for 8 h. **(E)** Terbinafine inhibits *S. pombe* growth. WT and *ltc1Δ* mutant cells expressing or not mCherry-D4H were diluted to OD 0.02 and incubated with 0.1 µg/ml terbinafine for 25 h in EMM-ALU. Afterward, OD was measured. Results represent three independent experiments. **(F)** Terbinafine does not display cytotoxic effect in *S. pombe*. Yeast cells were treated with 0.1 µg/ml terbinafine for 16 h at 25°C. Cell viability was evaluated by flow cytometry upon propidium iodide (PI) staining. **(G)** Microtubule depolymerization has no effect on mCherry-D4H distribution and relocalization. Cells expressing mCherry-D4H and tagged α-tubulin (GFP-Atb2) were incubated in the presence of DMSO (1%), CK-666 (500 µM), or metenzimidazoleazol-2-yl-carbamate (MBC 25 μg/ml), or were pretreated with MBC for 15 min and subsequently treated with CK-666 for 1 h. Arp2/3 inhibition resulted in relocalization of mCherry-D4H signal from the PM toward the cell interior independently of microtubule integrity. Microtubule depolymerization did not trigger relocalization of mCherry-D4H. Error bars show SD. Scale bars, 2 µm.

**Figure 1.**
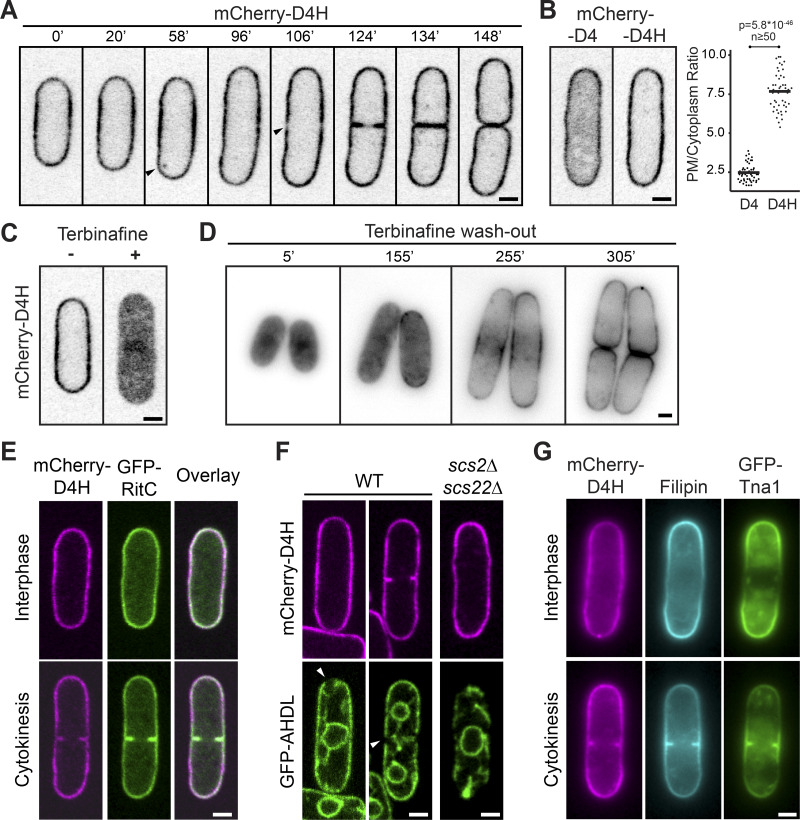
**The D4H sterol bio-sensor labels the plasma membrane in fission yeast cells. (A)** mCherry-D4H distribution during interphase growth in *S. pombe* cells. The arrowheads point to an internal dot at 58’ and clearance of D4H from the predivisional site at 106’. Time in minutes. **(B)** Localization of D4H and D4 sterol biosensors in vegetative cells. The plot on the right shows the PM to cytoplasm fluorescence ratio. Horizontal bars indicate the mean. **(C)** mCherry-D4H distribution in cells treated with the sterol biosynthesis inhibitor terbinafine for 16 h. **(D)** Recovery of mCherry-D4H PM localization upon wash-out of terbinafine after 16 h treatment. **(E)** mCherry-D4H colocalizes with the PM marker GFP-RitC. **(F)** mCherry-D4H decorates the PM, not the ER marked with GFP-AHDL in WT and *scs2Δ scs22Δ* mutants deficient in ER-PM attachment. Arrowheads point to zones of PM devoid of ER. **(G)** Distinct distribution of mCherry-D4H and filipin staining in cells also coexpressing GFP-Tna1. Scale bars, 2 µm.

To visualize sterol distribution in live fission yeast, we integrated mCherry-D4H as single copy under control of the constitutive actin promoter (*p^act1^*). Cells expressing this construct showed no growth problems or morphological defects (Fig. S1, A and B). D4H localized to the cell periphery throughout the cell cycle, with occasional occurrence of one or a few internal dots (Fig. 1 A and Video 1). The unmutagenized mCherry-D4 probe also localized to the cell cortex, but exhibited threefold lower cortex-to-cytosol signal ratio, consistent with its lower sterol affinity (Fig. 1 B). Treatment of cells with the squalene epoxidase inhibitor terbinafine or the lanosterol demethylase inhibitor ketoconazole, both of which block an early step in sterol synthesis, led to D4H delocalization to the cytosol, confirming the probe’s sterol-binding specificity (Fig. 1 C and Fig. S1, C and D). Note that terbinafine treatment was cytostatic and did not lead to substantial cell death (Fig. S1, E and F). Restart of sterol biosynthesis upon terbinafine removal led to D4H return to the cortex, interestingly populating first cell poles and division sites (Fig. 1 D). We conclude that D4H reports on the distribution of sterol-rich membranes in yeast cells.

**Video 1.** **mCherry-D4H distribution during vegetative growth.** Spinning-disk confocal time-lapse of mCherry-D4H during the interphase growth in *S. pombe*. The video is sped up 1,200-fold. Timing starts from the time the cells are placed on the agarose pad.

To probe whether D4H was at the PM or in the cortical ER, we coexpressed mCherry-D4H with the PM marker GFP-RitC (Bendezú et al., 2015) and ER marker GFP-AHDL. GFP-RitC fully colocalized with mCherry-D4H (Fig. 1 E). Consistent with close ER-PM apposition, GFP-AHDL and mCherry-D4H also showed extensive colocalization, but specific regions at cell tips and the division plane labeled by D4H were devoid of ER (Fig. 1 F). Moreover, in mutants lacking VAP family ER-PM tethers (*scs2Δ scs22Δ*), in which the ER is largely detached from the PM (Zhang et al., 2012), mCherry-D4H remained at the cell periphery (Fig. 1 F). Thus, D4H localizes to the PM, in line with the known high concentration of sterols in this compartment.

Interestingly, D4H intensity varied along the PM, from homogeneous to slight depletion at growing cell poles and clearance at the division site shortly before cell division (Fig. 1 A). This distribution is distinct from that of filipin, which is enriched at cell poles and division site (Fig. 1 G; Wachtler et al., 2003). The difference in D4H and filipin distributions may arise from distinct sterol distribution or different sterol accessibility in the inner and outer PM leaflets. D4H localization was also distinct from that of GFP-Tna1, a multipass transmembrane protein proposed to mark sterol-rich membrane domains (Makushok et al., 2016).

### Arp2/3 inhibition results in intracellular sterol accumulation

**Figure 2.**
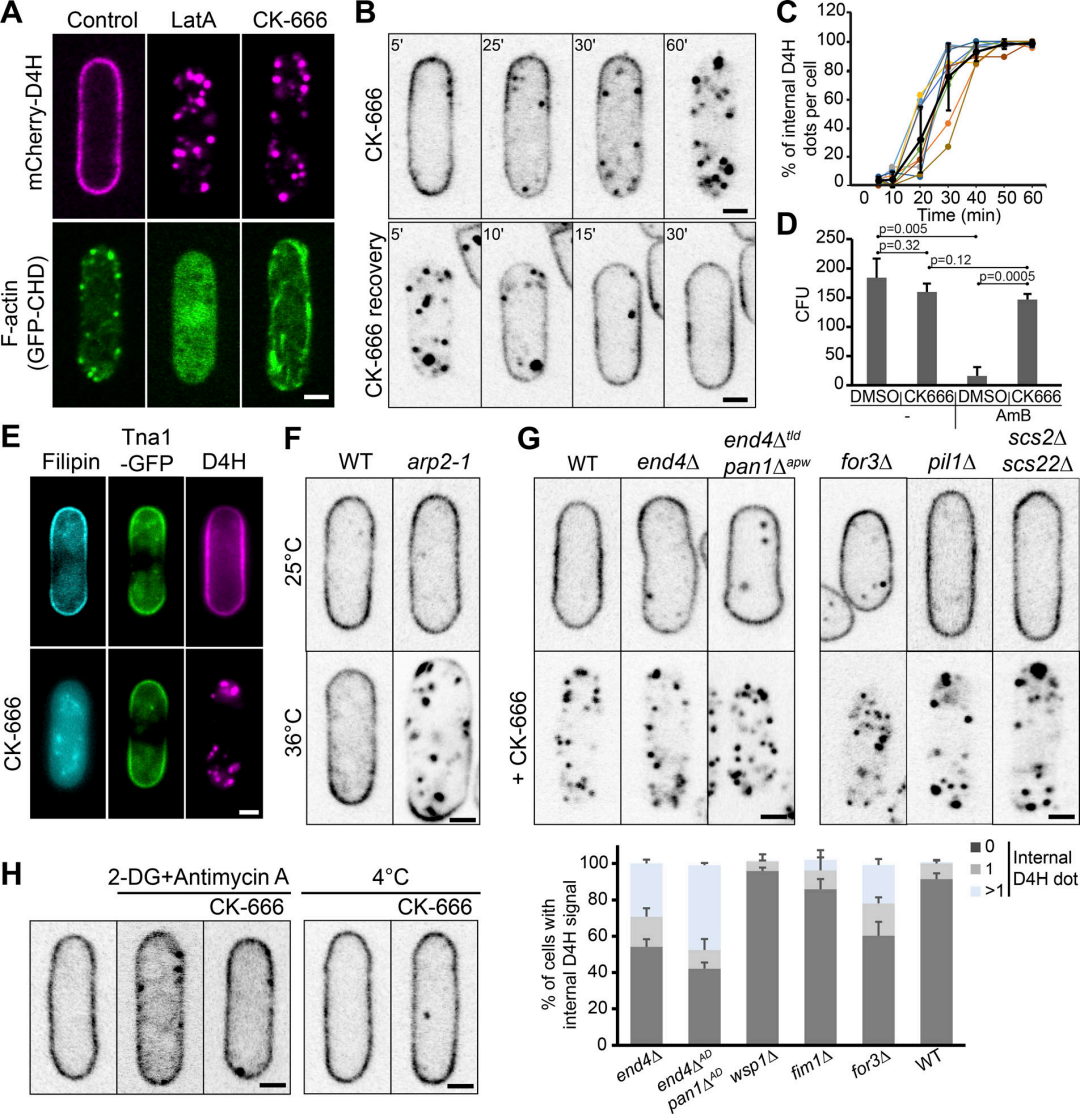
**Internalization of sterols upon inhibition of Arp2/3 activity. (A)** Localization of mCherry-D4H and F-actin marker GFP–calponin homology domain (CHD) upon depolymerization of F-actin by latrunculin A (LatA, 200 µM) or Arp2/3 inhibition by CK-666 (500 µM). **(B)** Time-lapse sequence of mCherry-D4H after CK-666 addition (upper panel) and wash-out after 1 h treatment (lower panel). **(C)** Quantification of the number of internal D4H-positive structures in CK-666–treated cells imaged every 5 min. Values are normalized to the maximal number of internal D4H dots in the z-stack. Error bars represent SD of seven independently tracked cells. **(D)** WT cells become resistant to AmB upon CK-666 treatment. The graph shows the viability of cells pretreated with CK-666 (or DMSO as control) for 1 h and then treated with a high dose (4 µg/ml) of AmB for another hour. *n* = 3 experiments. **(E)** Loss of filipin staining, but not PM GFP-Tna1 after 1 h CK-666 treatment. **(F)** mCherry-D4H in *arp2-1* mutant cells grown at 25°C or 36°C for 6 h. **(G)** mCherry-D4H in selected endocytic (*end4Δ, end4^Δtld^ pan1^Δapw^*), actin cable (*for3Δ*), eisosome (*pil1Δ*), and ER-PM attachment (*scs2Δ scs22Δ*) mutants treated or not with CK-666 for 1 h. The bottom graph displays the number internal dots in untreated cells in z-stacks (n ≥ 30 cells per strain). **(H)** mCherry-D4H relocalization upon energy depletion. Left: Cells were treated for 1 h with deoxyglucose (DG) and antimycin to deplete energy, and then incubated for 1 h more with CK-666. Right: Cells were precooled on ice for 30 min and then treated with CK-666 for 1 h on ice. Error bars show SD. Scale bars, 2 µm. CFU, colony-forming units.

We unexpectedly discovered that depolymerization of F-actin by Latrunculin A caused D4H loss from the PM and intracellular accumulation as discrete dots (Fig. 2 A). Specific Arp2/3 inhibition with CK-666 had the same effect (Fig. 2 A and Video 2; Nolen et al., 2009). CK-666 led to transient D4H enrichment at cell poles, appearance of a weak diffuse signal, formation of D4H-positive structures within 15 min, and clearance from the PM within 30–60 min (Fig. 2, B and C). In agreement with PM sterol reduction, cells pretreated with CK-666 for 1 h became resistant to amphotericin B (AmB), an ergosterol-binding antifungal that induces the formation of pores in sterol-rich membranes (Fig. 2 D). Moreover, the PM of these cells did not stain with filipin (Fig. 2 E). Thus Arp2/3 inhibition leads to strong reduction in PM sterol levels. The effects of CK-666 were fully reversible: 30–60 min after wash-out, D4H again decorated the PM (Fig. 2 B, lower panel; and Video 3). Arp2/3 inhibition by using the *arp2-1* temperature-sensitive allele yielded similar accumulation of D4H internal signal (Fig. 2 F). By contrast, D4H remained at the PM upon formin For3 deletion, though this mutant exhibited more internal dots than WT in untreated conditions (Fig. 2 G). Microtubule depolymerization had no effect on D4H distribution (Fig. S1 G). These results indicate a crucial role for F-actin assembly by Arp2/3 in sterol distribution.

**Video 2.** **mCherry-D4H relocalization upon CK-666 treatment.** Spinning-disk confocal time-lapse of mCherry-D4H after treatment with 500 µM CK-666. The video starts 4 min after CK-666 addition and is sped up 1,200-fold.

**Video 3.** **Recovery of mCherry-D4H cortical localization upon removal CK-666.** Spinning-disk confocal time-lapse of mCherry-D4H distribution recovery after wash-out of CK-666. The video starts 6 min after wash-out and is sped up 600-fold.

Because Arp2/3 inhibition blocks clathrin-mediated endocytosis in yeast, we probed whether inhibition of endocytosis by other means also provokes sterol internalization (Fig. 2 G). However, cells with moderate defects in endocytic patch dynamics, lacking *wsp1* or *fim1* (Lee et al., 2000; Sirotkin et al., 2005; Skau and Kovar, 2010), and cells with severely impaired endocytosis, lacking the adaptor protein End4 or carrying End4 and Pan1 alleles unable to bind actin (*end4^Δtld^ pan1^Δapw^*; Chen and Pollard, 2013; Iwaki et al., 2004), retained D4H at the PM and showed no or only moderate numbers of internal D4H-positive structures. Disrupting Arp2/3 in these mutants led to D4H movement to the cell interior as in WT cells. Thus, sterol redistribution is not caused by a block in clathrin-mediated endocytosis, but by inhibition of another Arp2/3 function.

**Figure S2.**
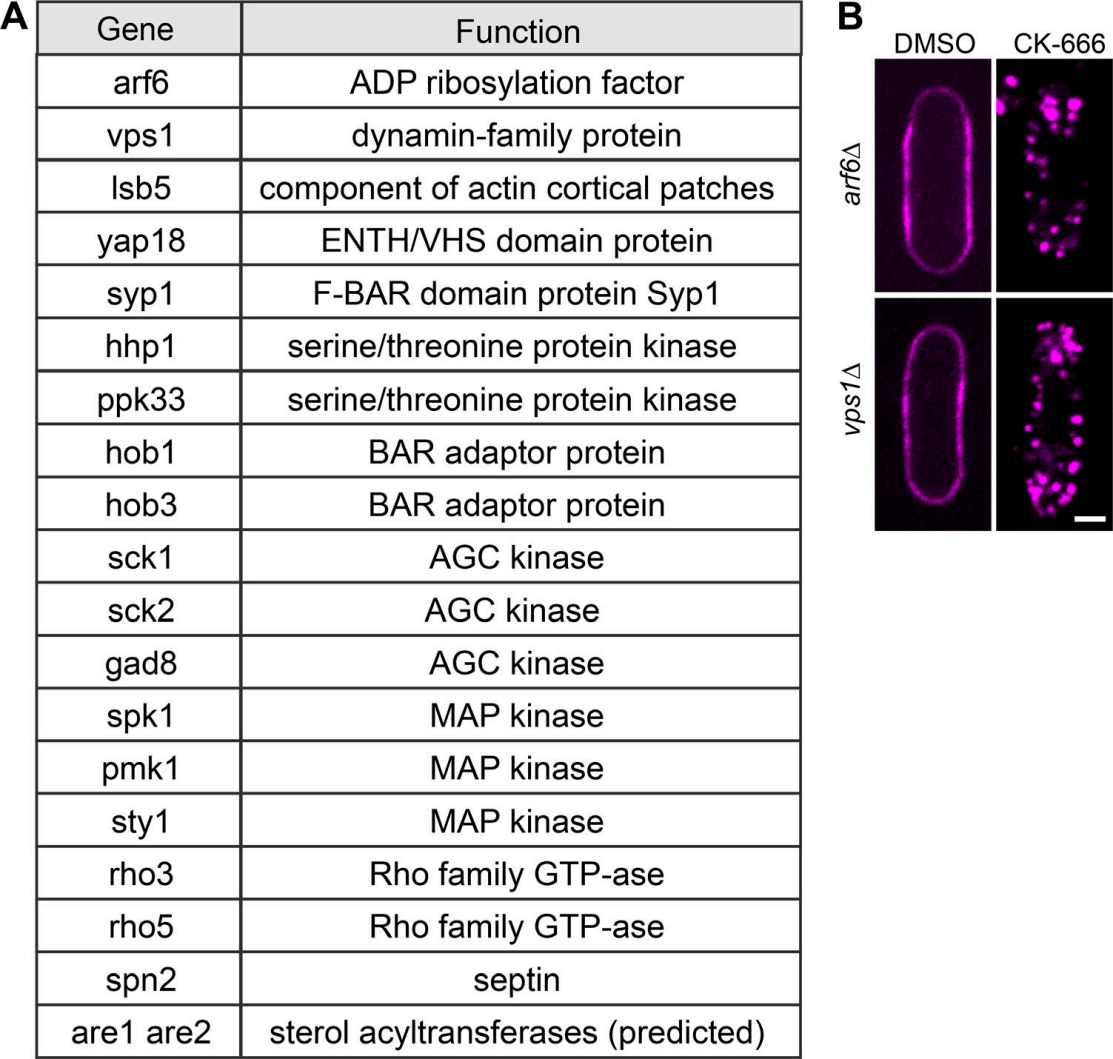
**Deletion strains tested for their ability to relocate mCherry-D4H upon CK-666 treatment. (A)** Listed deletion strains expressing mCherry-D4H were treated with CK-666 for 1 h and evaluated by spinning disc microscopy. All strains were capable of internalizing mCherry-D4H. **(B)** The deletion of *arf6* and *vps1*, encoding proteins predicted to be involved in nonclathrin mediated endocytosis, does not block mCherry-D4H internalization. *arf6Δ* and *vps1Δ* mutants expressing mCherry-D4H were incubated in the presence of 500 µM CK-666 for 1 h and evaluated by spinning disk microscopy. Both strains translocated the sterol biosensor D4H upon the treatment. Scale bar, 2 µm.

**Figure S3.**
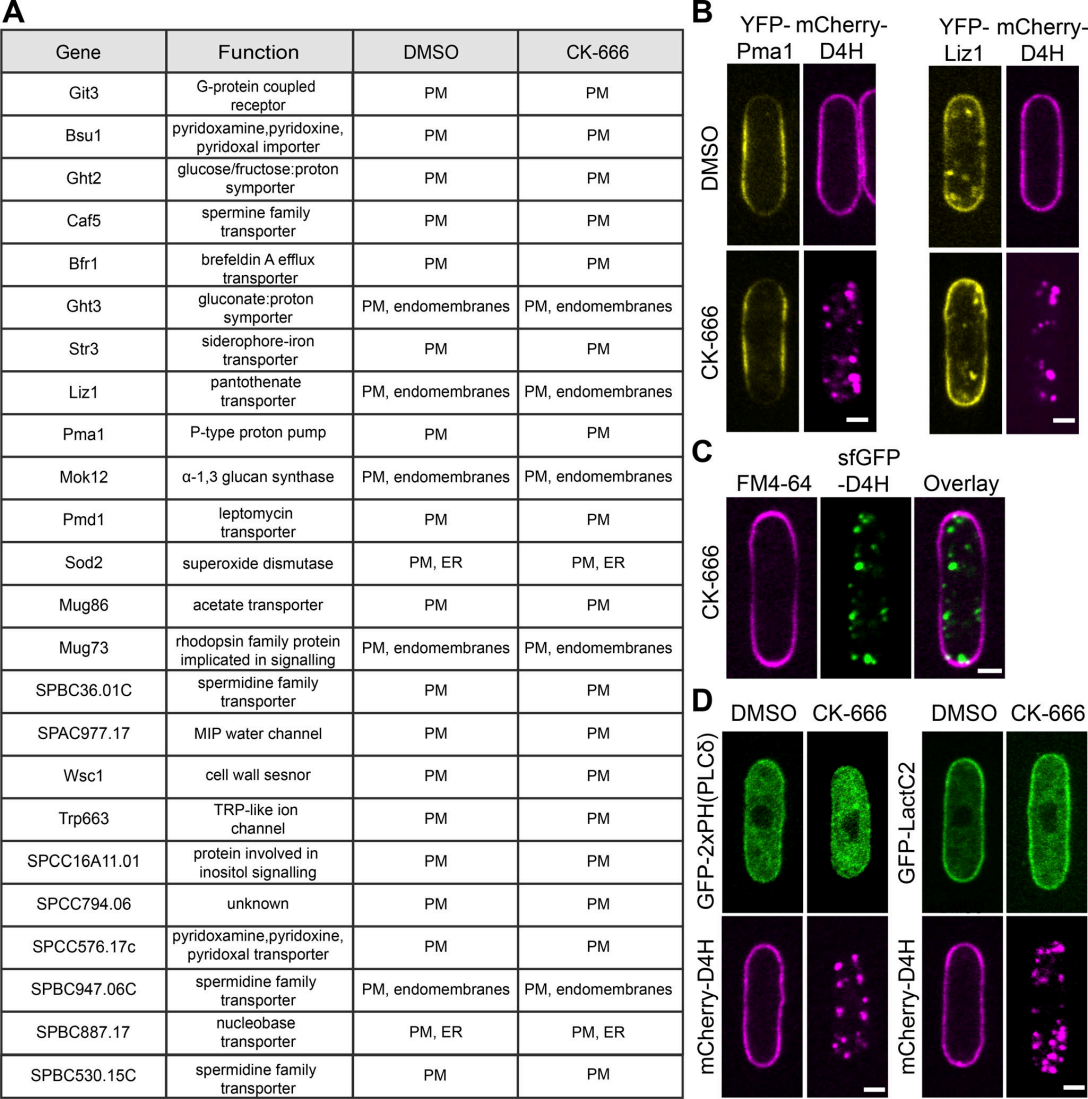
**List of YFP-tagged transmembrane proteins evaluated for colocalization with mCherry-D4H positive structures. (A)** *S. pombe* cells coexpressing mCherry-D4H, which were treated with 500 µM CK-666 or DMSO as control for 1 h at 25°C. Localization of D4H and YFP-tagged TM proteins was evaluated by spinning disk microscopy. In all cases, D4H localized to the PM in DMSO-treated cells and to internal dots in CK-666–treated ones. TM proteins could be broadly assigned to two classes: those exclusively at the PM in both conditions, and those decorating both PM and internal structures (endomembranes) in both conditions. Upon strong overexpression, several PM-localized TM proteins also decorated internal structures. In the second class, internal signals showed variable extents of colocalization with D4H dots upon CK-666 treatment. Notably, however, none of the TM proteins was exclusively at the PM in DMSO-treated cells and in internal structures upon CK-666 treatment. We also conducted further in-depth analysis by time-lapse imaging of a couple of TM proteins of the second class (Ght3, Liz1), with the aim to capture cointernalizing signals from the PM. However, although colocalization was observed on dynamic internal structures, we did not capture a single convincing event of internalization from the PM. We conclude that the internal signal of these (and likely other) TM proteins was present before CK-666 treatment, and may be partly due to the natural transit of these proteins through the secretory pathway. **(B)** Images of two exemplary TM proteins YFP-Pma1 localizing exclusively to PM and YFP-Liz1 localizing to PM and internal structures. Scale bar, 2 µm. **(C)** FM4-64 does not internalize in cells treated with CK-666. **(D)** Biosensors for phosphatidylinositol 4,5-bisphosphate (PI(4,5)P_2_) or phosphatidylserine (PS) are not internalized upon CK-666 treatment. Cells expressing mCherry-D4H and either a PI(4,5)P_2_ biosensor (2xPH_(PLCδ)_) or a PS biosensor (LactC2) were treated with 500 µM CK-666 for 1 h. These probes did not colocalize with D4H-positive internal structures. Scale bar, 2 µm.

We entertained the possibility that sterols may be internalized through a noncanonical actin-independent endocytic pathway. However, deleting homologues of genes involved in nonclathrin-mediated endocytosis in mammalian cells (Doherty and McMahon, 2009), such as dynamin (*vps1*) or *arf6*, did not block CK-666–induced D4H internal movement (Fig. S2). From a screen of over 24 transmembrane proteins at the PM, we were also unable to identify any cointernalizing with D4H, though some decorated internal structures in addition to the PM even in untreated conditions (Fig. S3, A and B). Notably, neither PM domain marker GFP-Tna1 nor lipophilic dye FM4-64 cointernalized with D4H upon treatment with CK-666 (Fig. 2 E and Fig. S3 C). PM loss and intracellular accumulation were also specific to the sterol lipids, as neither GFP-2xPH_(PLC∂)_ nor GFP-LactC2, which bind phosphatidylinositol bisphosphate (PIP_2_) and phosphatidylserine (PS ), respectively, internalized like D4H upon CK-666 treatment (Fig. S3 D). We conclude that sterol movement from the PM is unlikely to involve direct membrane endocytosis.

We also probed for a possible role of eisosomes, plasma membrane invaginations present in fungi and other walled eukaryotes, with suggested enrichment in sterol (Grossmann et al., 2007; Kabeche et al., 2015b). However, deletion of the major eisosome structural component Pil1 did not block CK-666–induced D4H relocalization (Fig. 2 G; Kabeche et al., 2011). The process was also independent of ER-PM contacts mediated by the VAP family tethers Scs2 and Scs22 (Fig. 2 G; Zhang et al., 2012). Interestingly, D4H translocation is an energy-requiring process since blocking ATP production with antimycin A or incubation of cells at 4°C blocked CK-666–induced D4H relocalization (Fig. 2 H). In summary, sterol movement to internal structures upon Arp2/3 inhibition is an endocytosis- and eisosome-independent, active process.

### Sterols accumulate within the endosomal compartment upon Arp2/3 inhibition

**Figure 3.**
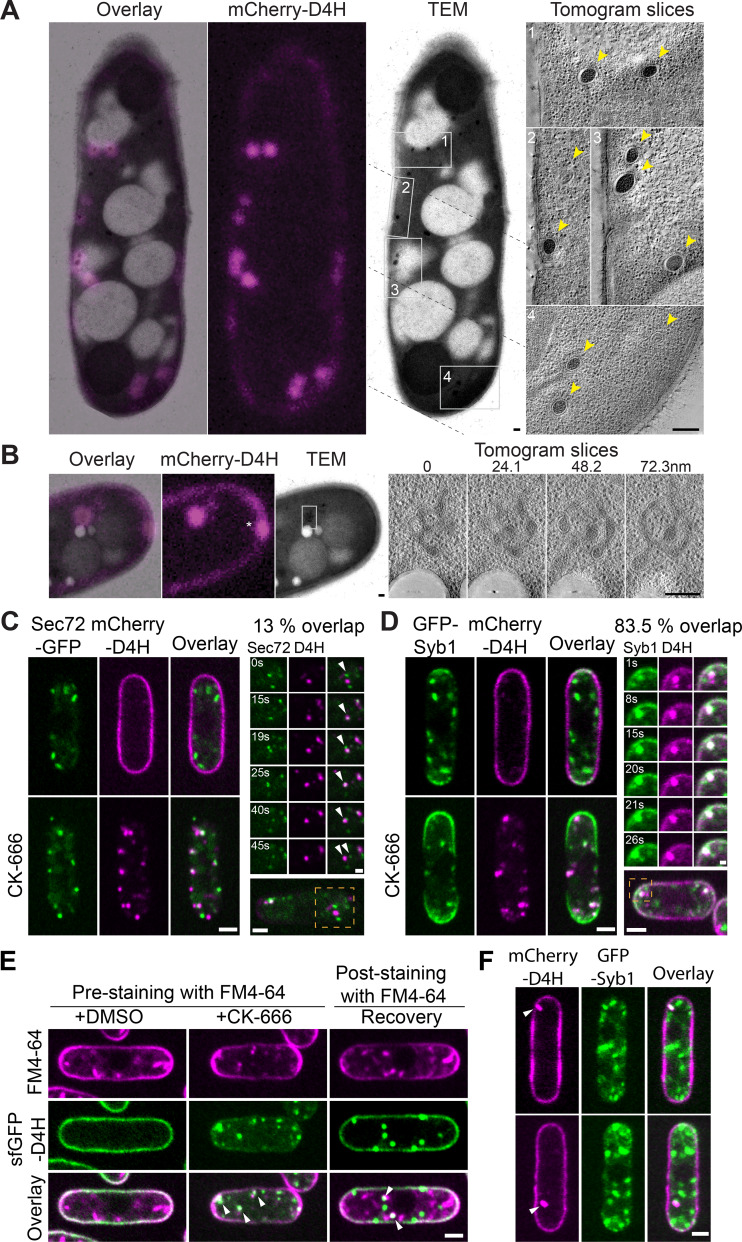
**Sterols are internalized to endosomes. (A and B)** Correlative TEM and epifluorescence (mCherry-D4H) images of a 300-nm section of mCherry-D4H–expressing cells treated with CK-666 for 45 min to 1 h. The overlay is shown on the left. In A, the TEM image is a composite of three micrographs (junctions indicated by dashed lines). Virtual sections through tomographic reconstruction of the four regions highlighted by gray boxes are shown on the right. In B, serial virtual sections at 24.1 nm distance through tomographic reconstruction of the boxed region are shown on the right. Yellow arrowheads point to D4H-positive spherical organelles. **(C and D)** Colocalization of mCherry-D4H and Sec72-GFP (C) or GFP-Syb1 (D) in cells with or without CK-666 for 1 h. An example time-lapse sequence is shown on the right. 13% of D4H internal dots overlapped with Sec72-GFP and 83.5% with GFP-Syb1 upon CK-666 treatment (n = 20 cells). **(E)** Colocalization of internal sfGFP-D4H (sfGFP-superfolder GFP) structures with internalized FM4-64 in cells prelabeled with FM4-64 and treated 30 min with CK-666 (left) or labeled with FM4-64 upon CK-666 wash-out (right). **(F)** Untreated WT interphase cells with internal mCherry-D4H signal, which colocalizes with GFP-Syb1. Scale bars, 200 nm in A and B; 1 µm in D, right panel; 0.5 µm in C, right panel; and 2 µm elsewhere.

We examined the nature of the internal D4H-positive structures after 45 min Arp2/3 inhibition by correlative light-electron microscopy (CLEM). We focused on cells that contained internal and PM signal, with the latter used to align the light and EM images. The majority of D4H internal signals (144 of 154 foci in 44 cells) corresponded to membrane-enclosed compartments, which we name sterol-rich compartments (STRIC). 77.3% of these were large, often electron-dense, almost spherical organelles with a diameter of 128.5 ± 26.9 nm (Fig. 3 A). The others overlapped with larger membrane organelles (18.7%; Fig. 3 B), possibly Golgi (see below), or PM invaginations (4%). We acquired tilt-series and reconstructed tomograms for 104 STRIC, which confirmed their vesicular nature (Fig. 3, A and B).

**Figure S4.**
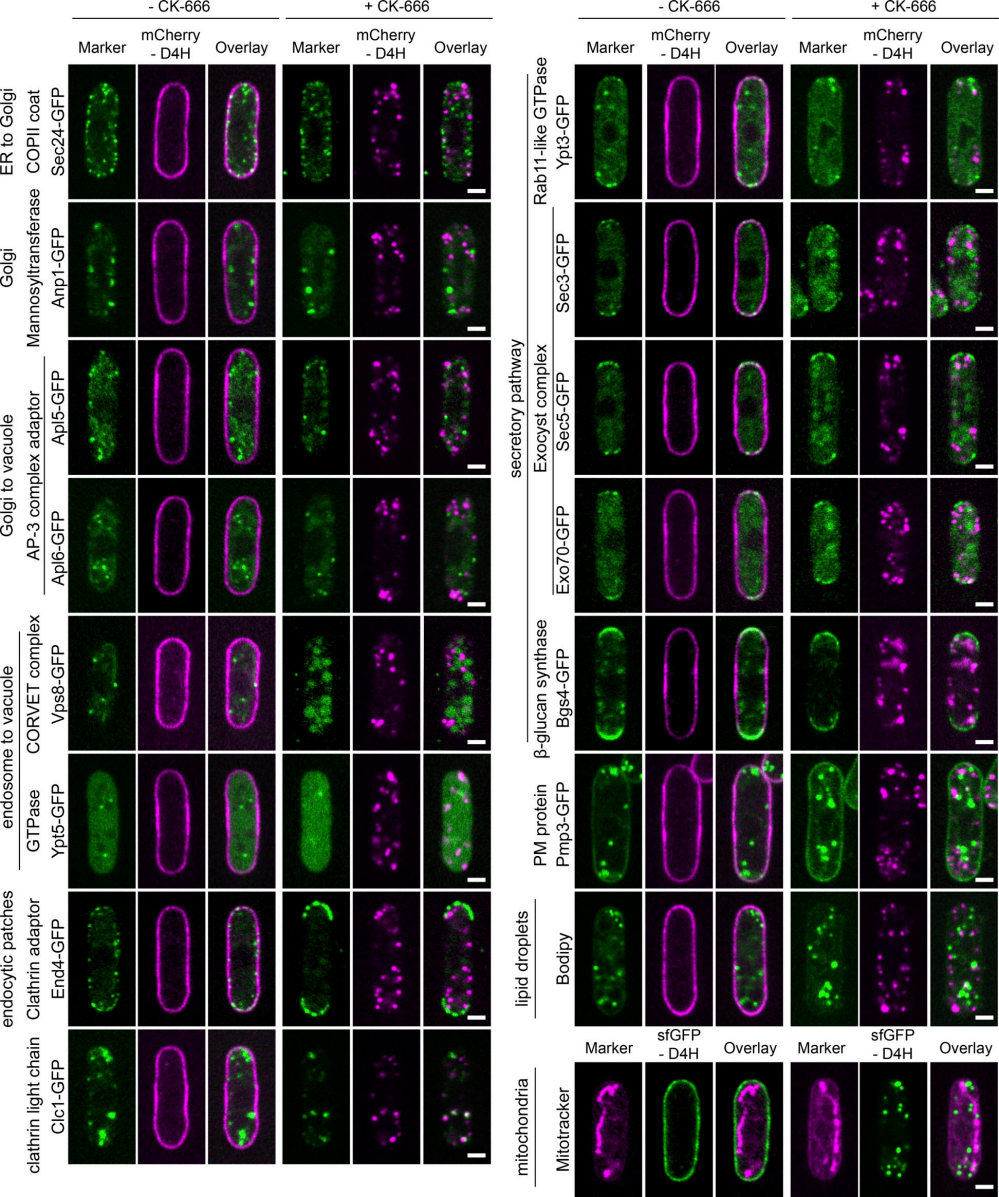
**Colocalization analysis of internal D4H signal with components of endo-membranes.** Marker proteins were tagged with GFP and expressed together with mCherry-D4H. Localization of both signals was compared in cells treated or not treated with CK-666. Sec24 is a COPII cargo receptor and transitional ER (tER) marker. Anp1 is a subunit of the mannosyltransferase complex that localizes to the early Golgi. Apl5 and Apl6 are subunits of the AP3 adaptor complex present in the TGN network. Vps8 is a subunit of the CORVET (Class C core endosome vacuole tethering) complex present in the prevacuolar compartment. Ypt5 is a Rab GTPase predicted to regulate the CORVET complex. End4 is a clathrin adaptor present at sites of endocytosis. Clc1 encodes the clathrin light chain, which localizes to endosomes/TGN and actin patches. Ypt3 is a Rab GTPase homologous to Rab11, localizing to secretory vesicles and the TGN. Sec3, Sec5, and Exo70 are exocyst components, localizing to secretory vesicles and the PM. Bgs4 is a 1,3-β-glucan synthase present at cell tips and within TGN. Pmp3 is a predicted PM protein, which also labels prominent, unknown internal structures. Bodipy labels lipid droplets. Mitotracker labels mitochondria. No colocalization was observed between any of these markers and D4H internal structures upon CK-666 treatment, except for Clc1, for which partial colocalization was observed. Scale bar, 2 µm.

**Figure S5.**
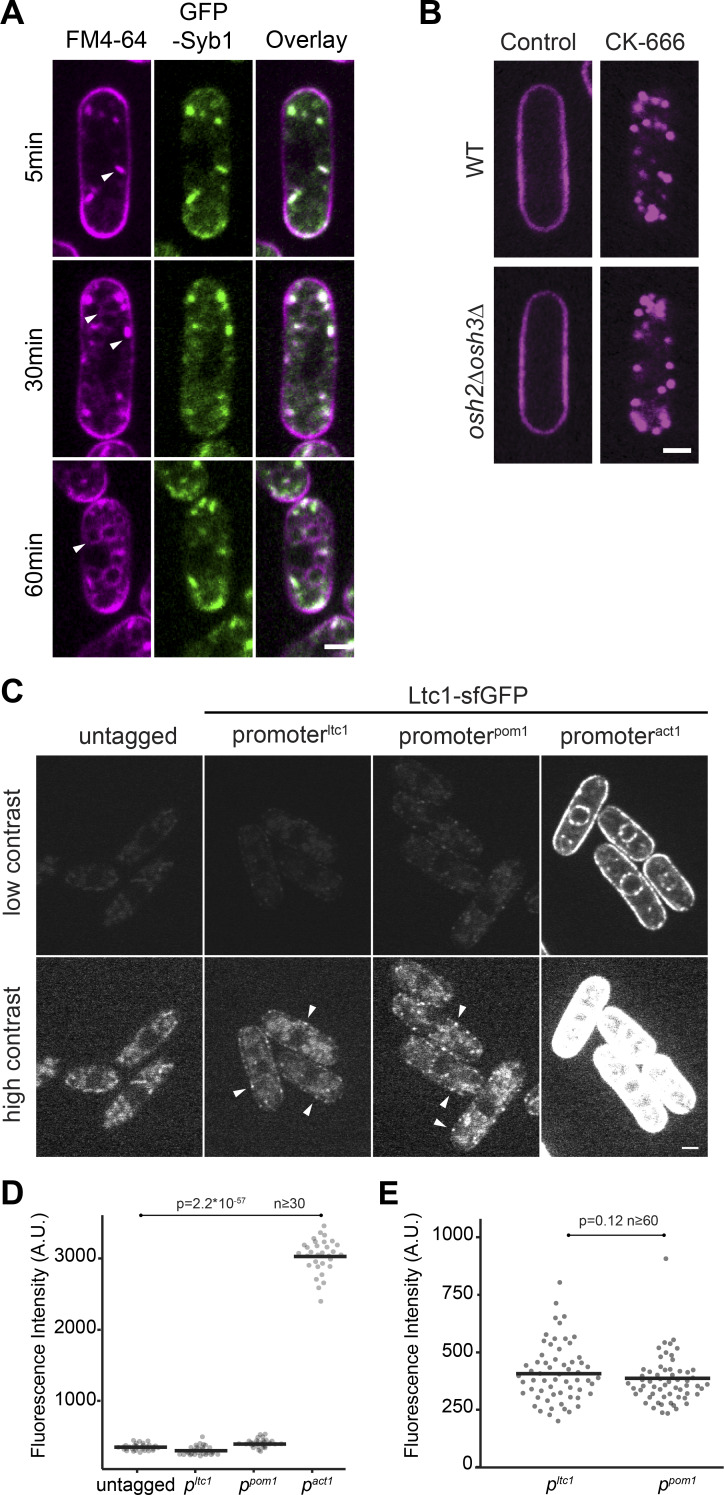
**Colocalization of GFP-Syb1 with internalized FM4-64, normal D4H distribution in *osh2Δ osh3Δ* double mutants and relative expression levels of Ltc1-sfGFP under native, *p^pom1^* and *p^act1^* promoters. (A)** GFP-Syb1 labels an endosomal compartment. Cells were incubated with FM-64 and dye distribution was evaluated after 5, 30, and 60 min after labeling. FM4-64 signal initially labels PM and endosomes, which strongly colocalize with GFP-Syb1. Endosomal and weak vacuolar staining can be observed 30 min after staining, and the dye reaches vacuoles within 60 min of incubation. Scale bar, 2 µm. **(B)** mCherry-D4H in WT and *osh2Δ osh3Δ* double mutant treated or not with CK-666 for 1 h. Scale bar, 2 µm. **(C)** Ltc1-sfGFP was expressed either under *ltc1* promoter at the native genomic locus, or under *p^pom1^* or *p^act1^* promoters. Untagged and Ltc1-sfGFP–expressing strains imaged in identical conditions are shown at the top. The images are contrasted optimally for either *p^act1^*-driven expression (top) or native expression (bottom). Arrowheads indicate sites of Ltc1 peripheral localization. Scale bar, 2 µm. **(D)** Quantification of total fluorescence in cells as in C. **(E)** Quantification of fluorescence in cortical punctae in cells as in C. A.U., arbitrary units.

To examine the STRIC identity, we labeled CK-666–treated cells expressing mCherry-D4H with a large panel of organellar markers. We did not detect significant colocalization with markers labeling ER exit sites, early Golgi, prevacuolar compartment, endocytic patches, post-Golgi vesicles, lipid droplets, or mitochondria (Fig. S4). By contrast, we detected occasional colocalization with the late Golgi/early endosome marker Sec72-GFP (Fig. 3 C): in time-lapse experiments, initially distinct D4H and Sec72 signals transiently overlapped for several time frames before dissociating again (Fig. 3 C and Video 4). This suggests that STRIC transiently associates with late secretory compartments, consistent with the CLEM data. Importantly, D4H strongly colocalized with the v-SNARE Synaptobrevin-like Syb1, a core component of the vesicle-PM fusion machinery, which resides on vesicles and is recycled from the PM (Fig. 3 D and Video 5). In untreated WT cells, Syb1 localizes to punctate structures that correspond to endosomal compartments (Edamatsu and Toyoshima, 2003; Gachet and Hyams, 2005), as shown by strong colocalization with the lipophilic dye FM4-64 internalized for 5 min (Fig. S5 A). A fraction of Syb1 also localizes to the PM, where it is trapped upon endocytosis arrest by CK-666 treatment. In these conditions, D4H colocalized with the residual internal Syb1-GFP signal. STRIC were also labeled by FM4-64 uptake if applied before CK-666 treatment or upon CK-666 wash-out (Fig. 3 E). We conclude that blocking Arp2/3 activity results in accumulation of sterols within the endosomal compartment.

**Video 4.** **Colocalization of mCherry-D4H with Sec72-GFP during treatment with CK-666.** Spinning-disk confocal time-lapse of cells coexpressing mCherry-D4H and Sec72-GFP after treatment with CK-666. The video is sped up 10-fold.

**Video 5.** **Colocalization of mCherry-D4H and GFP-Syb1 during treatment with CK-666.** Spinning-disk confocal time-lapse of mCherry-D4H and GFP-Syb1 distribution after treatment with CK-666. The video is sped up 10-fold.

Because internal D4H dots were also observed in untreated cells (Fig. 1 A and Video 1; 20.8 ± 2.5% of all cells), we asked whether these also correspond to STRIC labeled by Syb1-GFP. Indeed, 32.6 ± 6% of these naturally forming D4H dots colocalized with Syb1 (Fig. 3 F). These observations suggest that STRIC also form in unperturbed cells, but are enriched upon Arp2/3 inhibition.

### Sterols travel between the PM and endosomes independently of the secretory pathway

**Figure 4.**
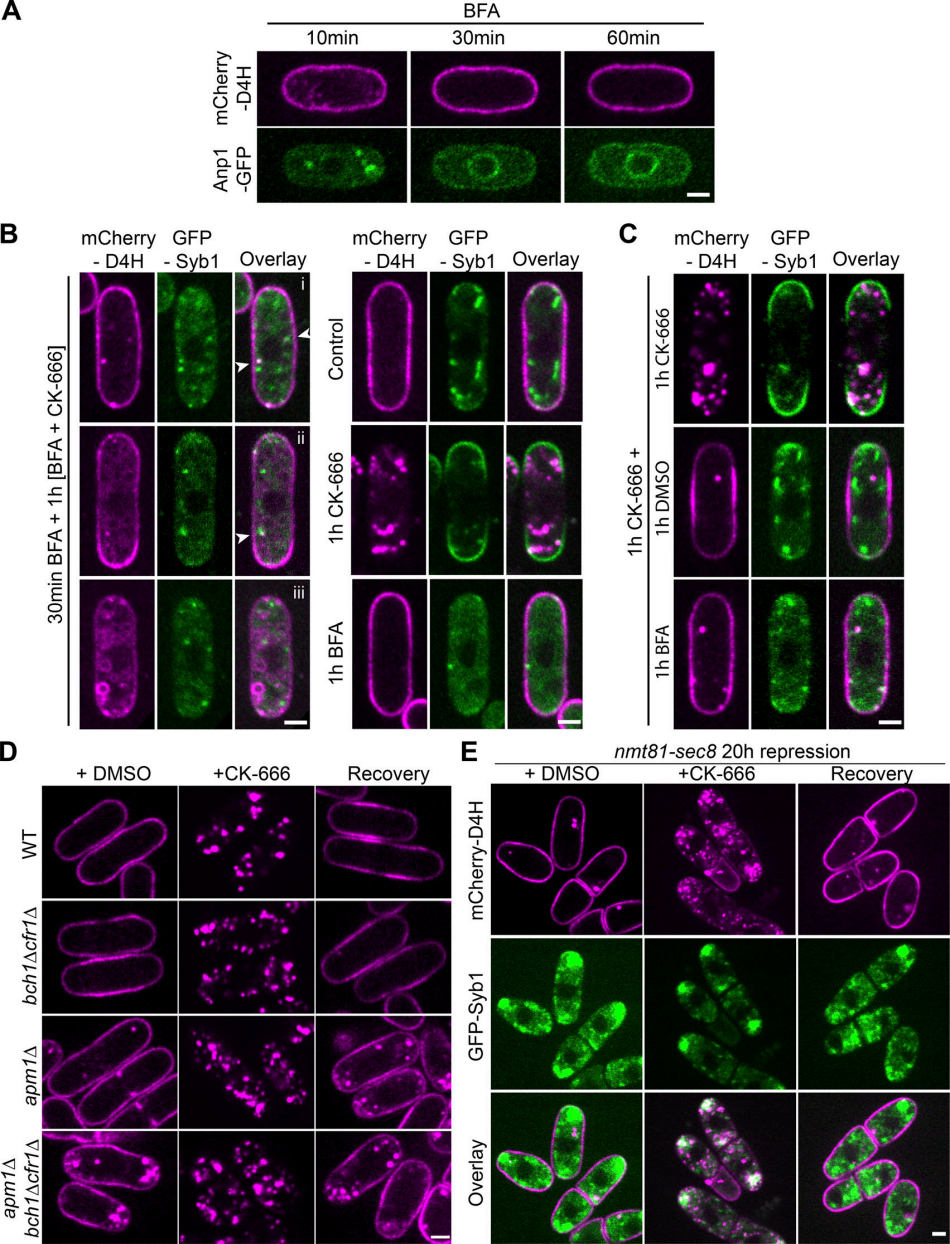
**Sterol transport is independent of vesicle trafficking. (A)** Treatment of cells with 300 µM BFA leads to collapse of Anp1-GFP–marked Golgi to the ER, but has no effect on mCherry-D4H distribution at the PM. **(B)** Cells pretreated with BFA for 30 min to induce Golgi collapse and then incubated in the presence of BFA and CK-666 to induce sterol internalization display three principal phenotypes: (i) mCherry-D4H at the PM with occasional internal dot-like structure that coincided with Syb1 (arrowheads), (ii) mCherry-D4H at the PM and internal haze, with dot like-structures that coincided with Syb1, (iii) weak mCherry-D4H at the PM with internal haze and vacuolar staining. The right panel shows control treatments. **(C)** Cells were treated for 1 h with CK-666, and then 1 h more with CK-666, DMSO, or BFA. **(D)** mCherry-D4H localization during steady-state growth (DMSO), upon 1 h CK-666 treatment, and after 1 h recovery in exomer-deficient mutants (*bch1Δ cfr1Δ*), mutants lacking the AP-1 adaptor complex subunit (*apm1Δ*), and triple mutant (*bch1Δ cfr1Δ apm1Δ)*. **(E)** mCherry-D4H localization during steady-state growth (DMSO), upon 1 h CK-666 treatment, and after 1 h recovery in cells in which *sec8* expression (under the control of the *p^nmt81^* promoter) has been repressed for 20 h. Scale bars, 2 µm.

To assess the importance of the secretory pathway in sterol movement to endosomes, we treated cells with Brefeldin A (BFA). BFA is a macrocyclic lactone that inhibits the Sec7-family of ARF exchange factors, leading to collapse of the Golgi (Peyroche et al., 1999). In *S. pombe*, BFA blocks secretion and leads to Golgi disappearance with concomitant ER accumulation (Turi et al., 1994). Indeed, BFA caused the redistribution of the early Golgi marker Anp1-GFP to the ER and the dispersion of most Syb1 signal, indicating loss of most or all ER-anterograde trafficking (Fig. 4, A and B). 1 h BFA treatment had no effect on D4H localization (Fig. 4 A). However, consistent with sterol trafficking through endosomes, in cells pretreated with BFA for 30 min to induce Syb1 dispersion, Arp2/3 inhibition largely failed to induce the formation of STRIC: the D4H signal either remained at the PM (Fig. 4 B, i) and/or showed an internal haze and/or vacuolar staining (Fig. 4 B, ii and iii). Interestingly, occasional internal dots (Fig. 4 B, i and ii) coincided with remaining Syb1-positive structures. These data indicate that sterol movement from the PM is strongly perturbed upon BFA treatment, with redirection of sterols to other endomembranes and residual transport to remaining Syb1-positive structures.

CK-666 wash-out leads to D4H return to the PM (Fig. 2 B and Video 3). Interestingly, addition of BFA at the time of CK-666 removal did not impair D4H signal recovery to the PM (Fig. 4 C). Trafficking from the endosome is regulated by the clathrin adaptor protein Apm1 (AP-1) and the exomer complex, and *apm1Δ*, and more strongly *apm1Δ exomerΔ*, accumulate post-Golgi vesicles (Hoya et al., 2017; Kita et al., 2004). Consistent with their aberrant endosomes, *apm1Δ* and *apm1Δ exomerΔ* triple mutants showed increased D4H internal signal (Fig. 4 D). However, CK-666 treatment led to D4H relocalization to endosomes as in WT cells, with recovery to the PM upon CK-666 wash-out to levels observed during steady-state growth (Fig. 4 D). The exocyst complex, which promotes secretory vesicle tethering to the PM, is critical for polarized secretion (Heider and Munson, 2012), and depletion of the essential subunit Sec8 leads to subapical accumulation of Syb1-positive organelles (Bendezú and Martin, 2011). Upon Sec8 depletion, D4H remained at the PM, relocalized to Syb1 compartments upon CK-666, and returned to the PM upon CK-666 wash-out, despite the subapical retention of Syb1 (Fig. 4 E). We conclude that, similar to retrograde transport from PM to endosomes, anterograde transport of sterols from the endosome to the PM is independent of the canonical vesicular trafficking machinery.

### Sterol distribution in sterol transport mutants

**Figure 5.**
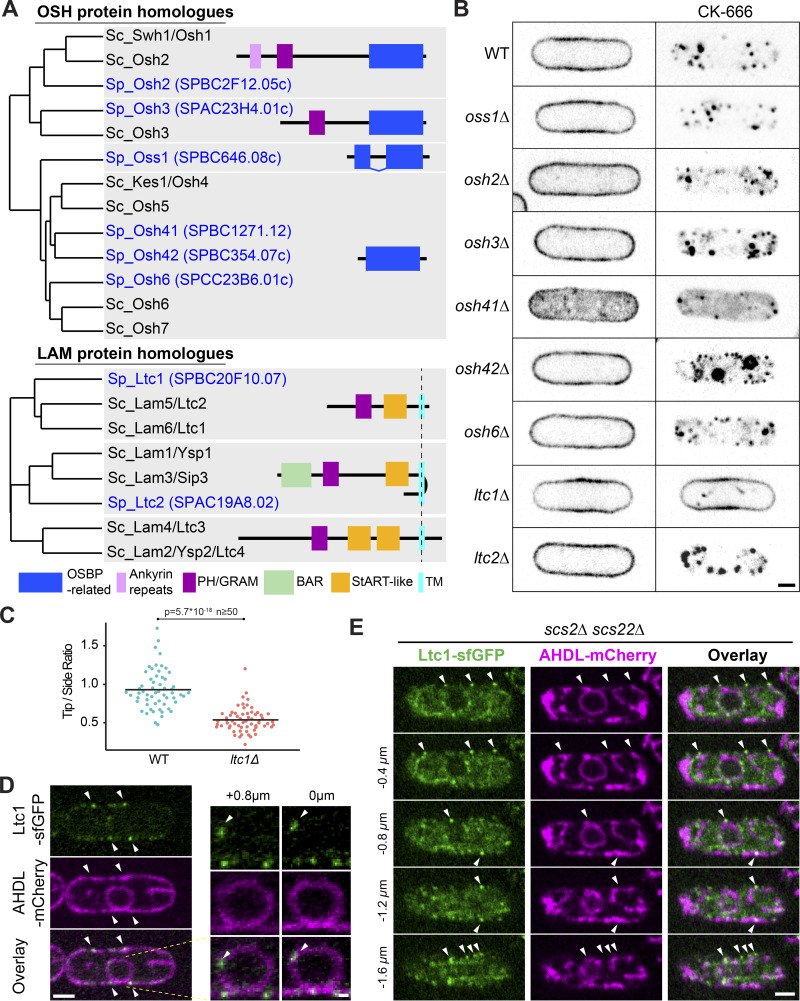
**Ltc1 is required for sterol internalization and localization to ER-PM contact sites. (A)** Neighbor-joining phylogenetic trees of *S. pombe* and *S. cerevisiae* OSH and LAM family proteins. The *S. pombe* genes were named in analogy to the *S. cerevisiae* names, with Osh = oxysterol-binding protein homologue, Oss1 = oxysterol-binding split-domain protein, and Ltc1/2 = lipid transfer at contact site. Protein domains identified by SMART are indicated (http://smart.embl-heidelberg.de/). **(B)** mCherry-D4H in OSH family or LAM family deletion mutants treated or not with CK-666 for 1 h. **(C)** Quantification of mCherry-D4H fluorescence intensity at cell tip versus cell side. Bars show the mean (n ≥ 50 cells). **(D)** Ltc1-GFP expressed from the native genomic locus localizes to cortical and internal dots (arrowheads) at the ER, labeled with mCherry-AHDL. Right panels show an enlargement of the nucleus. **(E)** Ltc1-GFP localizes to residual ER-PM contacts (arrowheads) in the *scs2Δ scs22Δ* mutant. Panel represents middle plane view and four subsequent planes at 0.4 µm distance. Scale bars, 0.5 µm in D, right panel, and 2 µm elsewhere.

Sterol trafficking may involve sterol-binding transporter proteins belonging to the ORP and LAM protein families (Luo et al., 2019). *S. pombe* encodes six ORP and two LAM proteins, which we named according to their phylogenetic relationship with *S. cerevisiae* homologues (Fig. 5 A). We generated deletions in each gene (Fig. 5 B). In most mutants, D4H localized correctly to the PM under standard growth conditions, with two exceptions (Fig. 5 B, left panel). First, *osh41Δ* cells showed only a weak PM signal, with significant D4H signal in the cytosol and on small internal structures. Second, *ltc1Δ* cells showed D4H at the PM, but the signal was depleted from cell poles (Fig. 5 C and Video 6). Because of described links of Osh2 and Osh3 with Arp2/3 activation in *S. cerevisiae* (Encinar Del Dedo et al., 2017), we also constructed a double *osh2Δ osh3Δ* mutant, which, however, showed normal D4H distribution (Fig. S5 B).

**Video 6.** **mCherry-D4H distribution during vegetative growth in *ltc1Δ* mutant.** Spinning-disk confocal time-lapse of mCherry-D4H during the interphase growth in *ltc1Δ*. The video is sped up 1,200-fold. Timing starts from the time the cells are placed on the agarose pad.

Upon CK-666 treatment, again most mutants were proficient in sterol movement to endosomes, including *osh41Δ* and *osh2Δ osh3Δ* (Fig. S5 B), with two exceptions. First, *osh42Δ* showed relocalization not only to endosomes but also to vacuoles (Fig. 5 B, right panel). Second, *ltc1Δ* completely blocked endosomal sterol enrichment, with D4H remaining PM-associated (Fig. 5 B, right panel; and Video 7). Below, we focused on the role of Ltc1 in retrograde sterol transport.

**Video 7.** **mCherry-D4H relocalization upon CK-666 treatment in *ltc1Δ* mutant.** Spinning-disk confocal time-lapse of mCherry-D4H distribution upon treatment with CK-666 in *ltc1Δ*. The video starts 4 min after CK-666 addition and is sped up 1,200-fold.

### Ltc1 localizes to ER-PM contact sites and regulates PM to endosome sterol flow

**Figure 6.**
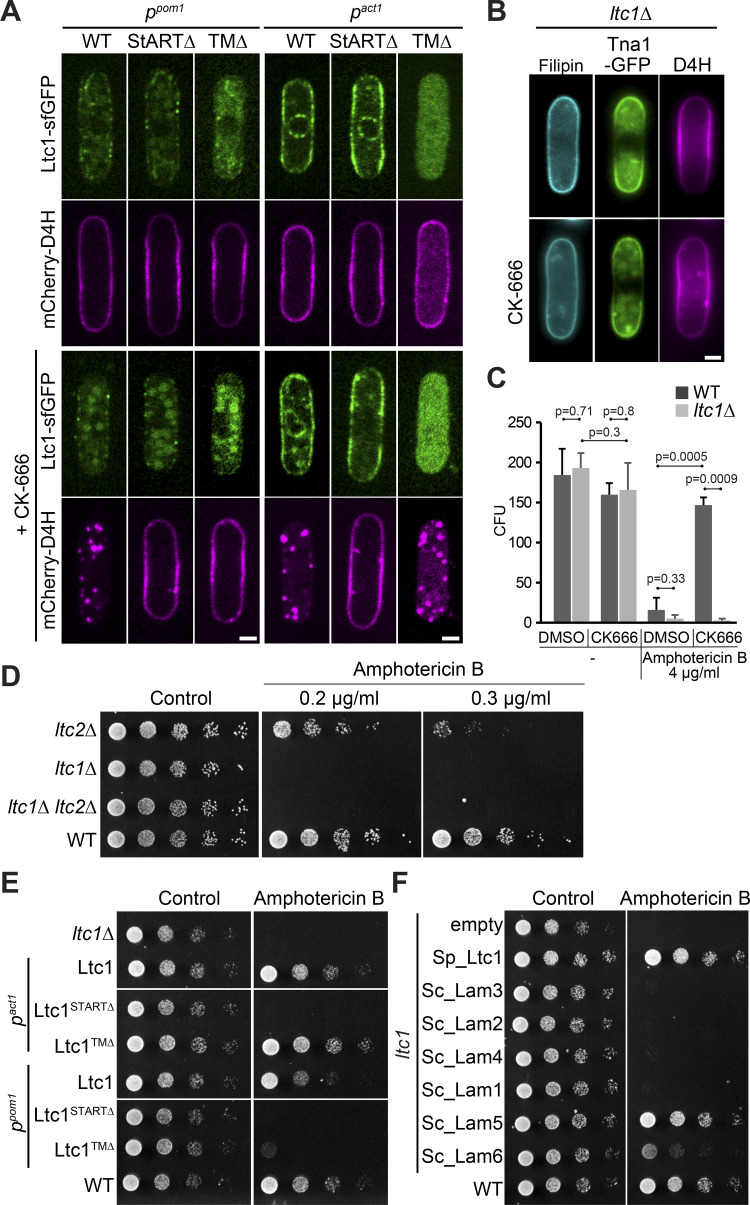
**Ltc1 StART-like domain is essential for retrograde sterol transfer. (A)** Ltc1-sfGFP and truncated alleles lacking StART-like or TM domain expressed under control of the weak *p^pom1^* promoter or strong *p^act1^* promoter in *ltc1Δ* cells, treated or not with CK-666 for 1 h. **(B)** Filipin remains at the PM of *ltc1Δ* cells treated with CK-666 for 1 h. **(C)** WT but not *ltc1Δ* cells become resistant to AmB during CK-666 treatment. WT data and methodology as in Fig 2 D. **(D)** Serial dilutions of WT and indicated mutants on EMM-ALU media containing AmB. **(E)** Serial dilutions of *ltc1Δ* strains expressing WT Ltc1 or truncated alleles under the control of *p^pom1^* and *p^act1^* on plates containing 0.2 µg/ml AmB. **(F)** Serial dilutions of *ltc1Δ* mutant expressing *S. cerevisiae* LAM proteins under the control of *p^act1^* on plate containing 0.2 µg/ml AmB. Scale bars, 2 µm.

C-terminally sfGFP-tagged Ltc1 expressed from the native genomic locus, Ltc1-sfGFP, localized to puncta principally at the cell cortex and occasionally in the cell interior, including the perinuclear ER (Fig. 5 D). Interestingly, in *scs2Δ scs22Δ* double mutants that exhibit fewer ER-PM contacts than WT cells, cortical Ltc1-sfGFP puncta formed at these residual contact sites (Fig. 5 E), indicating Ltc1 marks VAP family–independent ER-PM contact sites. Ltc1-sfGFP overexpression under the control of the strong actin (*p^act1^*) promoter led to labeling of the entire ER (Fig. 6 and Fig. S5 C), indicating that Ltc1 is an ER-resident protein. We conclude that Ltc1 resides in the ER and is enriched at ER-PM contact sites.

Ltc1 exhibits three notable domains: a PH-like GRAM domain, a StART-like domain predicted to bind sterols, and a C-terminal transmembrane (TM) domain (Fig. 5 A). To determine the importance of these domains for Ltc1 localization and function, we expressed in *ltc1Δ* cells full-length Ltc1 as well as mutants lacking these domains (Ltc1^GRAMΔ^, Ltc1^StARTΔ^, and Ltc1^TMΔ^). These constructs were expressed not under native promoter but under the control of either the strong *p^act1^* or the weaker *p^pom1^* promoter (Vjestica et al., 2019). Expression of full-length Ltc1 under *p^pom1^* control was at levels similar to the native locus and localized to puncta that were not affected by CK-666 treatment (Fig. 6 A; and Fig. S5, C–E). Ltc1^StARTΔ^ localization was indistinguishable from WT. Ltc1^TMΔ^ localized to the cytosol. Ltc1^GRAMΔ^ was likely unstable as no or very little signal was detected, and so was not studied further. To test functionality of the truncations, we treated cells with CK-666 and assessed D4H localization. At low expression levels, D4H movement to the cell interior was observed with full-length Ltc1 but with none of the truncation mutants (Fig. 6 A, left panel). At high expression levels, both full-length Ltc1 and the *ltc1^TMΔ^* allele rescued D4H internalization (Fig. 6 A, right panel), suggesting that localization to ER-PM contacts primarily serves to increase local concentration of the protein. Ltc1^TMΔ^ also promoted an increase in cytosolic D4H in untreated cells. These data are consistent with the view that sterols move from the PM to endosomes through direct transfer by Ltc1 StART-like domain and that the main role of the TM domain is to increase the concentration of Ltc1 at sites of sterol transport.

### Sterol flow contributes to plasma membrane homeostasis

In line with the D4H retention at the PM in *ltc1Δ* treated with CK-666, these cells retained PM filipin staining and did not become resistant to high AmB concentrations (Fig. 6, B and C). Even in steady-state conditions, *ltc1Δ* cells showed enhanced AmB sensitivity, which was complemented by overexpression of Ltc1^TMΔ^, but not any other truncation mutant (Fig. 6, D and E). We used AmB sensitivity to test for functional homologues among LAM-family proteins, which showed that *S. cerevisiae* Lam5 and Lam6 are able to restore AmB resistance to *ltc1Δ* cells (Fig. 6 F). This is consistent with phylogenetic proximity of Ltc1 with these two *S. cerevisiae* LAM family proteins (Fig. 5 A). Thus, *ltc1Δ* has excess sterols at the PM not only upon CK-666 treatment, but also during steady-state growth.

**Figure 7.**
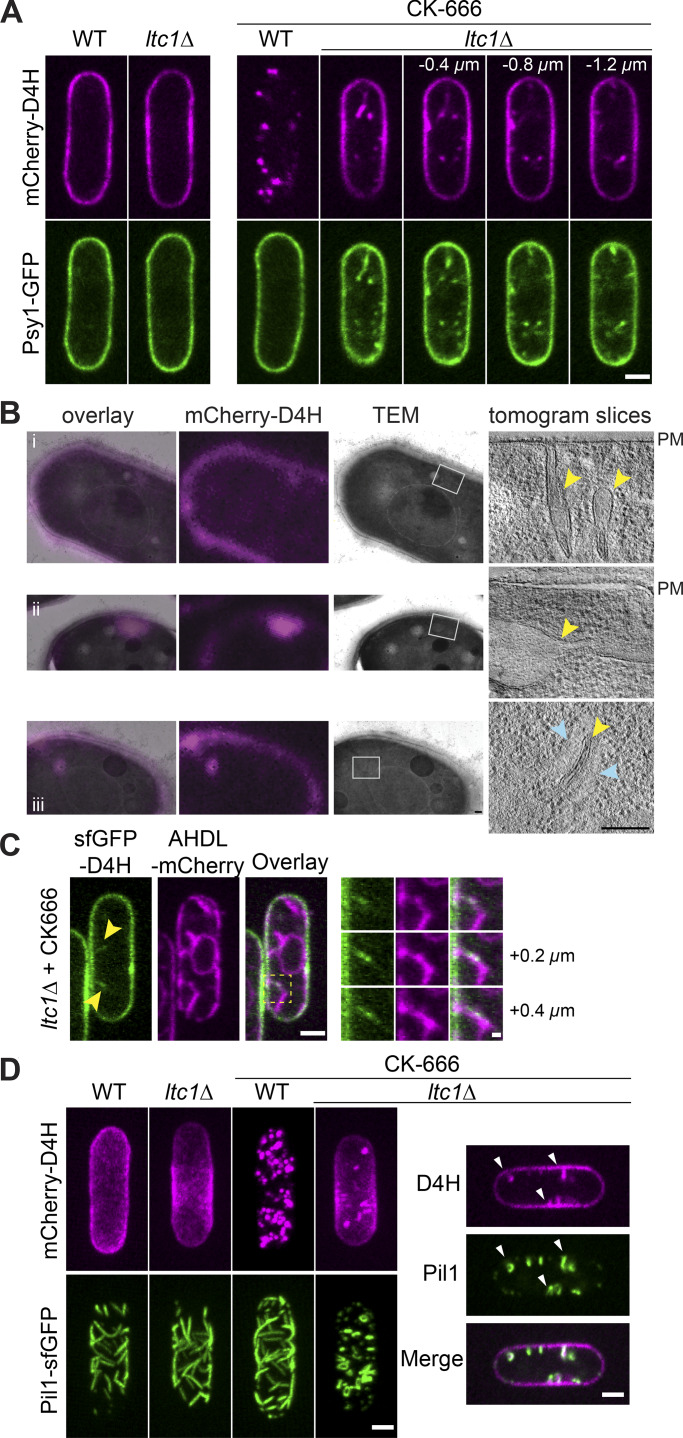
**Deficiency in retrograde sterol transfer leads to large PM invaginations. (A)** Internal mCherry-D4H signal in CK-666–treated *ltc1Δ* cells form deep invaginations colabeled by the PM t-SNARE GFP-Psy1. **(B)** Correlative TEM and epifluorescence (mCherry-D4H) images of a 300-nm section of mCherry-D4H–expressing *ltc1Δ* cells treated with CK-666 for 45 min to 1 h. The overlay is shown on the left. Virtual sections of tomographic reconstruction of the regions highlighted by gray boxes are shown on the right. Three examples show (i) invaginations from the PM, (ii) large internal bulging region, and (iii) tube surrounded by ER (blue arrowheads). Yellow arrowheads point to D4H-positive organelles. **(C)** PM invaginations labeled by sfGFP-D4H in CK-666–treated *ltc1Δ* mutant are surrounded by ER marked by mCherry-AHDL. An enlargement of the boxed region is shown over three z-stacks on the right. **(D)** PM invaginations labeled by sfGFP-D4H in CK-666–treated *ltc1Δ* form at eisosomes, marked by Pil1. In WT cells, Pil1 and D4H do not colocalize. In *ltc1Δ* cells, Pil1 appears to surround the D4H signal on PM invaginations. Images on the left are maximum projections of 0.4-µm-spaced z-planes. Scale bars, 200 nm in B; 0.5 µm in C, right panel; and 2 µm elsewhere.

Remarkably, *ltc1Δ* cells not only did not move sterols to endosomes but also formed long PM invaginations upon CK-666 treatment. Residual internal D4H structures observed in CK-666–treated *ltc1Δ* cells were not vesicular as in WT cells but elongated and connected to the PM, as shown by confocal sectioning. These PM invaginations were decorated by resident PM proteins, such as the t-SNARE Psy1 (Fig. 7 A). CLEM cells showed that out of 32 internal fluorescence signals, 22 corresponded to membrane tubes or sheets (Fig. 7 B, i and iii), occasionally forming larger bulges or more complex structures (Fig. 7 B, ii). In four cases, connection of the tube to the PM was captured within the section (Fig. 7 B, i). In several instances, these tubes were in very close proximity to ER structures, with an average closest distance of 15.8 ± 4.3 nm (*n* = 7; Fig. 7 B, iii), similar to cortical ER-PM distances measured in *S. cerevisiae* (Hoffmann et al., 2019; West et al., 2011). We also observed two signals overlapping with grooves at the PM. We did not observe any overlap between the D4H signal and large electron-dense vesicles as in WT cells, though two signals overlapped with smaller vesicles. Interestingly, we also found close contact between D4H-labeled invaginations and AHDL-labeled ER by light microscopy (Fig. 7 C). We conclude that blocking both endocytosis and sterol flow to endosomes leads to an excess of PM, which now forms extended ER-proximal internal invaginations.

The invaginations were marked by Pil1, suggesting that they arise from eisosome expansion (Fig. 7 D). Pil1-marked eisosomes appeared normal in *ltc1Δ*, forming extended furrows in the plane of the membrane as in WT cells. However, CK-666 addition caused a reorganization of Pil1 structures, which lost their planar organization and instead protruded inwards (Fig. 7 D). CK-666 addition to WT cells only caused a modest reorganization of eisosomes, which now also formed at cell poles, likely due to growth arrest (Fig. 7 D). Together, these data show that simultaneous block of endocytosis and sterol transfer from the PM leads to excess PM, which leads to aberrant enlargement and alteration of eisosomes.

## Discussion

In this study, we have used a genetically encoded fluorescent probe, D4H, to detect sterol-rich membranes in live cells, which revealed an intracellular sterol flux elicited upon Arp2/3 inhibition: sterols are carried from the PM to endosomes, likely through the ER, with the help of the lipid transfer protein Ltc1, and back to the PM independently of classical vesicular transport.

### The D4H biosensor gives novel insights into sterol distribution in cellular membranes

Because D4H is genetically encoded and nontoxic, it makes a highly valuable sterol biosensor. In unperturbed cells, it primarily labels the PM, but also occasionally intracellular TGN/endosomes. Surprisingly, the sterol distribution detected by D4H at the PM is distinct from that of filipin, which has been widely used to describe sterol-rich domains in fungi. D4H is fairly homogeneous along the PM or even depleted at growth sites, which contrasts with the labeling of cell poles and division sites by filipin. Because filipin is applied from the cell outside and D4H is expressed inside, this discrepancy may suggest distinct sterol distributions in the outer and inner PM leaflets, with a higher concentration of sterols in the outer leaflet at sites of polarized growth. Indeed, other approaches have also suggested an asymmetry in sterol distribution between the two yeast PM leaflets (Solanko et al., 2018). Alternatively, filipin and D4H localization may be influenced by differences in sterol accessibility, for instance due to complex formation with other lipids (e.g., sphingolipids). The distinct filipin staining pattern may also reflect cell wall permeability since both tips and division sites are places of strong cell wall remodeling. A clear advantage of D4H is its ability to noninvasively track sterol-rich endo-membranes (containing >20 mol%) in live cells, which filipin’s toxicity does not permit. We note that the transmembrane protein Tna1, which was previously proposed to label sterol-rich domains (Makushok et al., 2016), also fails to track sterol on endo-membranes.

D4H revealed alterations in sterol distribution for the deletion of three predicted sterol-binding LTPs: single deletions of *osh41*, *osh42*, and *ltc1* all affected D4H localization, though in distinct ways. While we have focused here on the role of Ltc1 in retrograde sterol flow from the PM, our observations suggest that Osh41 may promote PM sterol levels during steady-state growth, and Osh42 may control sterol flow away from vacuoles. Osh41 and Osh42 are related to *S. cerevisiae* Osh4, which binds sterols in vitro (de Saint-Jean et al., 2011; Im et al., 2005; Schulz et al., 2009) and is proposed to promote ER to PM sterol transport by using the counter-gradient of phosphatidylinositol-4-phosphate (PI4P; Moser von Filseck et al., 2015), although in vivo data suggest a role in membrane sterol organization rather than sterol transport (Georgiev et al., 2011; Quon et al., 2018; Raychaudhuri et al., 2006). Our initial observations in *S. pombe* suggest that Osh41 may promote the anterograde movement of sterol from the ER to endosome, but not from the endosome to the PM, as *osh41Δ* cells efficiently recovered D4H at the PM after CK-666 wash-out (unpublished data). These observations demonstrate that D4H is a highly valuable tool to study the intracellular trafficking of sterols.

### Retrograde trafficking of sterols from the plasma membrane to endosomes

We discovered that inhibition of Arp2/3-mediated actin assembly triggers the movement of D4H from the PM to intracellular STRICs. These organelles are labeled by late Golgi/endosome markers, such as the Arf GEF Sec72 and the v-SNARE Synaptobrevin-like Syb1, as well as by the internalized FM4-64 dye. The morphology and electron density of the STRIC is in fact highly reminiscent of that of secretory vesicles, suggesting these may represent a recycling/sorting compartment destined to the PM. The overlay of D4H and Syb1 signals in exocyst-depleted cells, which accumulate 100-nm-diameter vesicles (Wang et al., 2002), further supports this view. Thus, D4H is relocalized to endosomes. Endosomal sterol enrichment occurs beyond fungi. In mammalian cells, the endosomal/lysosomal compartment plays an important role during sterol redistribution to the PM after uptake of low density lipoprotein (LDL)–derived cholesterol (Kanerva et al., 2013). In plant cells, sterols were also shown to accumulate in endosomes upon disruption of the actin cytoskeleton (Grebe et al., 2003). Thus, actin-dependent endosome-PM sterol exchange may be a conserved feature of sterol trafficking.

Importantly, the sterol-rich endosomes are distinct from early endocytic vesicles. Four lines of evidence show that sterols do not travel from the PM to endosomes in endocytic vesicles. First, sterol flow to endomembranes occurs after inhibition of Arp2/3 with CK-666, which blocks clathrin-mediated endocytosis in yeast (Gachet and Hyams, 2005; Galletta and Cooper, 2009). Second, this flow occurs normally in cells in which endocytosis is genetically impaired. Third, despite an extensive screen, we did not identify any PM marker, whether peripherally associated or transmembrane, that cointernalizes with D4H. Fourth, sterol movement is not affected by mutants predicted to affect membrane sculpting (Bin/Amphyphysin/Rvs protein or dynamin mutants), but is blocked in absence of the previously uncharacterized LAM family protein Ltc1. We conclude that sterols do not reach the endosome through an endocytic route, but are transported between membranes by lipid transfer proteins.

**Figure 8.**
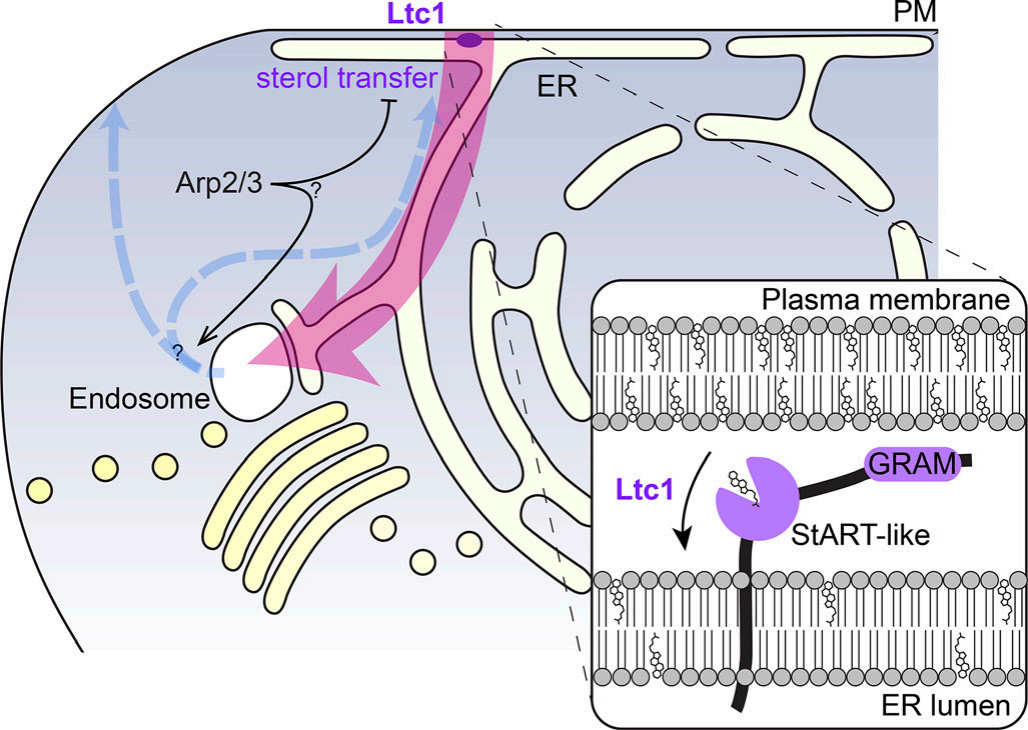
**Hypothetical model for the flux of sterols in fission yeast cells.** Sterol molecules are transported in the cell by ways orthogonal to vesicular trafficking. The StART-like domain Ltc1 is necessary for sterol retrograde transport from the PM, likely to the ER. Sterols then move in an anterograde manner that bypasses the Golgi to endosomes. From the endosome, sterols return to the PM independently of vesicular secretion, either through the ER or more directly. Arp2/3-dependent F-actin assembly actively promotes the transport and/or retention of sterols to the PM.

What route do sterols follow from the PM to endosomes? Because D4H labels the PM and endosomes, but no other endo-membrane, and because sterol depletion from the PM is inefficient in the absence of the Syb1 compartment, the simplest explanation would be a direct transport from PM to endosomes. However, several considerations suggest that the transport route may be less direct. First, Ltc1 is an ER resident that localizes to ER-PM contact sites, suggesting that it promotes sterol transport between these two organelles. Second, in *ltc1Δ*, we observed tight ER contacts with expanded regions of the PM, suggesting that these contacts exist independently of successful sterol transport. Third, we can make some rough quantitative estimates of sterol flow from PM to endosomes, which suggest there is not enough space on endosomes: these estimates make the assumption that the changes of D4H sterol detection observed here are solely or mainly due to changes in sterol membrane concentration, rather than other events that would alter D4H accessibility to sterols. Let us consider a 10-µm-long yeast cell with 30 mol% sterol at the PM (detectable by both D4 and D4H), which drop to 20 mol% (just below D4H detection levels) as they flow to 50 endosomes (upper bound of our observations) in 1 h. With a conversion of 30 mol% to 150 sterol molecules per 240 nm^2^ of inner membrane leaflet (Hofsäss et al., 2003), ∼25 million sterol molecules are moved within 1 h. An estimate of 5,000 Ltc1 molecules per cell (Carpy et al., 2014) leads to minimal rates of ∼1.4 sterols/s, which is not far to that measured for an isolated StART-like domain in vitro (0.84 sterols/s; Jentsch et al., 2018). With these numbers, while the surface of the PM is ∼125 µm^2^, the total endosomal surface is only ∼2.5 µm^2^, i.e., 50-fold lower. Thus, even a massive 50 mol% sterol concentration increase on endosomes would only account for 10% of the moved sterols, suggesting that a pool of internalized sterols is also present at other locations than endosomes. We hypothesize that these sterol molecules travel through the ER, in which, given its much larger membrane surface, the sterols are diluted to a concentration below the D4H detection limit. In support of this view, BFA treatment, which strongly depletes endosomes, leads to the redirection of sterols to other endo-membranes, including the vacuole, and likely backflow to the PM, preventing complete D4H depletion from the PM. Thus, a possible route involves sterols flowing from the PM to the ER and then from the ER to endosomes (Fig. 8). Direct demonstration that sterols transit through the ER will be required to establish this route with certainty.

### Ltc1 is essential for retrograde sterol transfer from the PM

The scenario described above implies at least two steps in PM to endosome transport: a retrograde movement between the PM and the ER, and an anterograde transport from the ER to endosomes. Three pieces of data indicate that Ltc1 mediates the first of these two steps. First, we provided several lines of evidence that the PM of *ltc1Δ* cells displays elevated sterol levels, even in unperturbed conditions. Second, Ltc1’s StART-like domain is essential for its function in retrograde sterol transport. Although we have not tested whether this domain directly binds sterols, the finding that the AmB sensitivity of *ltc1Δ* can be rescued by *S. cerevisiae* Lam5 and partly Lam6, which not only bind sterol but also shuttle it between membranes in vitro (Gatta et al., 2015; Murley et al., 2015), is supportive of Ltc1 directly binding and transferring sterol molecules. Third, Ltc1 localizes principally to VAP family–independent ER-PM contact sites, at the ideal location to promote sterol retrograde flow. This localization is, however, not strictly required, as overexpression of cytosolic Ltc1^TMΔ^ restores sterol flow to *ltc1Δ* cells, similar to what was observed for Lam2/Ysp2 in *S. cerevisiae* (Gatta et al., 2015). This suggests that Ltc1’s localization at ER-PM contacts serves to increase local concentration and boost the efficiency of sterol transport. Fourth, D4H depletion from cell poles in *ltc1Δ* is consistent with the view that sterol mobilization from the ER-proximal PM at cell sides fails in this mutant, leading to sterol accumulation at cell sides. We conclude that Ltc1 is very likely to mediate PM to ER retrograde sterol transfer (Fig. 8).

The second step, ER to endosome transport, likely also involves direct sterol transfer at ER-endosome contacts. This is suggested from the observation that sterols could transfer to residual Syb1-compartments after BFA treatment that led to complete Golgi collapse. It is also consistent with previous data on sterol anterograde transport being independent of the canonical vesicular trafficking system (Baumann et al., 2005; Heino et al., 2000; Urbani and Simoni, 1990). ER-endosome contacts are well established in mammalian cells, where they function in cholesterol transfer, endosome positioning, fission, and signaling (Raiborg et al., 2015). For instance, STARD3 and ORP-family ORP1L contribute to sterol trafficking from the ER to the endosome (Eden et al., 2016; Wilhelm et al., 2017). In yeast, ER-endosome contacts have not been studied but are suggested from the Lam5 localization in *S. cerevisiae* (Weill et al., 2018). In *S. pombe*, the LTP responsible for ER-to-endosome transfer is unknown. Because, except for *ltc1Δ*, no single OSH or LAM family deletion failed to accumulate sterols in endosomes, none of these proteins participates as sole player. The misdirection of D4H to vacuoles in *osh42Δ* mutants suggests Osh42 could promote transfer to endosomes, perhaps in conjunction to its paralog Osh41. Osh2 and Osh3, which facilitate Arp2/3-mediated actin polymerization in *S. cerevisiae* (Encinar Del Dedo et al., 2017), could potentially play a role, though double mutants did not reveal any clear phenotype. Ltc1 could also potentially mediate sterol transfer at endosomes, as it localizes to additional locations than ER-PM contacts, and is functionally rescued by *S. cerevisiae* Lam5. In this case, Ltc1 may represent an evolutionarily ancient LTP, structurally similar to, and functionally rescued by, Lam5, but whose localization and function in retrograde sterol transport from the PM are reminiscent of those of Lam2/Ysp2 (Gatta et al., 2015), for which there is no *S. pombe* orthologue. One important question for the future will be to understand what defines the directionality of the sterol transfer.

One striking consequence of blocking both endocytosis by Arp2/3 inhibition and sterol retrograde flow by *ltc1* deletion is the formation of very long PM invaginations. We hypothesize that ongoing secretion and/or transfer of other lipids in these conditions leads to an expansion of the PM, which acquires an excessive surface. This surface increase manifests itself through an enlargement of eisosomes, which change shape, forming long invaginations. This observation is in line with eisosomes buffering PM size also in other stressful conditions (Kabeche et al., 2015a). Whether and how these long invaginations contribute to signaling remains to be established, but it is interesting that they appear to be in tight contact with the ER. Close association of eisosomes with cortical ER is also observed in untreated cells (Ng et al., 2020). We also note that eisosomes are not required for PM-to-endosome sterol transfer, as D4H accumulated in endosomes upon CK-666 treatment in *pil1Δ* as in WT cells. We hypothesize that, in the presence of Ltc1, the retrograde flow of sterol, which may involve the transport of over 25 million sterol molecules at a rate of at least 7,000/s, within the rate estimation in *S. cerevisiae* (Dittman and Menon, 2017), compensates for the secretion-mediated increase in membrane. Thus, retrograde sterol flow regulates not only the PM sterol concentration but also PM surface homeostasis.

### Endosome-to-PM transport of sterols is independent of the vesicular trafficking pathway

Like PM-to-endosome sterol transport, we found that the return of sterols to the PM is independent of the canonical vesicular trafficking pathway. Indeed, disruption of the clathrin AP-1 adaptor Apm1 and the exomer complex, which promote exit from the endosome, or the exocyst complex, which is essential for the tethering of secretory vesicles with the PM, does not prevent the return of sterols from the endosome to the PM.

These findings are consistent with earlier finding in *S. cerevisiae* that sterol anterograde trafficking occurs largely normally in temperature-sensitive mutants of the yeast secretory pathway (Baumann et al., 2005). This earlier study tracked sterol by different means, extracting PM sterols with methyl-*β*-cyclodextrin, which only extracts <0.5% of total cellular sterol. Thus, the two studies, performed in distinct organisms and with different methodologies, come to the common conclusion that cellular sterol transport occurs by means independent of vesicular trafficking. One open question is the specific mechanism of sterol transfer from endosomes to the PM, which could either use a route back through the ER, or a more direct transfer from endosomes to the PM (Fig. 8).

### Why does Arp2/3 inhibition lead to sterol accumulation in endosomes?

Our data clearly demonstrate that Arp2/3 function plays an important role in sterol flows in the cell. Up to now, the only ascribed function of Arp2/3 in yeast was to promote endocytic membrane invagination. We found that mutants with defects in endocytic uptake did not cause D4H endosomal accumulation and reacted in the same way as WT to Arp2/3 inhibition. These data lead to the conclusion that sterol accumulation on endosomes is not a direct consequence of endocytosis failure, and demonstrate a novel, endocytosis-independent role for Arp2/3-mediated actin assembly in yeast cells.

This raises the question of the specific role of Arp2/3 in sterol flux. The observation that acute inhibition of Arp2/3 function leads to massive accumulation of sterols in endosomes suggests the two nonmutually exclusive possibilities that Arp2/3 inhibition blocks an existing flux leading to retention of sterols in endosomes or promotes the movement of sterols from the PM. In the first scenario, Arp2/3 may act at the endosome to promote anterograde sterol transfer, such that its inhibition leads to a retention of sterols in endosomes. In support of this, Arp2/3 is well-established to localize to endosomes and play numerous roles in cargo sorting and membrane fission in mammalian cells (Simonetti and Cullen, 2019). The spherical shape of STRIC after CK-666 treatment, which contrasts with the more varied shapes of Syb1-positive organelles in untreated cells, also suggests that Arp2/3-dependent actin nucleation may contribute to membrane deformation on the endosome in yeast cells. In agreement with sterol flux occurring in steady-state growth conditions, we observed occasional D4H-labeled endosomes in unperturbed cells. In the second scenario, Arp2/3 may act at the PM to limit the retrograde transfer by Ltc1, such that its inhibition facilitates PM to endosome flow. For instance, inhibition may change PM properties such as membrane tension, which may induce sterol transfer by Ltc1. We note, however, that walled fungal cells are not believed to harbor an actin cortex similar to that present in the nonwalled metazoan cell (Chugh and Paluch, 2018). It will be fascinating to discover this novel function of Arp2/3 in sterol homeostasis.

## Materials and methods

### Strains, growth conditions, and drug treatment

*S. pombe* strains used in this study are listed in Table S1. Standard genetic methods and growth conditions were used. Cells were grown in Edinburgh minimal medium supplemented with amino acids (EMM-ALU) or in rich YE5S at 25°C. Temperature-sensitive *arp2-1* mutant was incubated for 6 h at 36°C before analysis.

The following inhibitors were used at indicated final concentration: 200 µM latrunculin A (in DMSO), 500 µM CK-666 (in DMSO), 25 µg/ml benzimidazole carbamate (MBC), 0.1 µg/ml terbinafine (in ethanol), and 300 µM BFA (in ethanol). AmB was added at a final concentration 0.2 µg/ml unless otherwise indicated. For energy depletion, cells were pelleted and dissolved in EMM-ALU containing 20 mM deoxyglucose and 10 µM antimycin B (in ethanol).

Latrunculin A was ordered from Enzo Life Sciences and FM4-64 from Thermo Fisher Scientific. All other chemicals were ordered from Merck.

For recovery from CK-666 treatment, cells were incubated with CK-666 for 1 h, washed twice with medium, and imaged on agarose pads immediately after washing.

For filipin staining, the drug was added at final concentration of 5 µg/ml from DMSO stock to the cells diluted in EMM-ALU. Cells were imaged live within a maximum of 5 min on glass slides.

For depletion of Sec8 from the *nmt81-*s*ec8* strain, precultures were grown in EMM-ALU lacking thiamine and then diluted to OD 0.025 in YE5S containing thiamine, as described (Bendezú and Martin, 2011). Cells were cultivated for an additional 20 h at 25°C before imaging.

For measurement of cell length and width, cells were stained with Calcofluor White. 1 µl of Calcofluor White stock (2 mg/ml) was added to 500 µl cells in EMM-ALU. Cells were harvested immediately, resuspended in residual medium, and visualized in DAPI channel. A minimum of 25 cells undergoing division was evaluated.

For visualization of FM-64 uptake, cells were stained with 8 µM FM-64 dye for 2.5 min in YE5S medium and then washed three times with YE5S before microscopy.

For generation of deletion mutants, WT strains were transformed with linearized deletion plasmids (based on the pFA6a backbone) containing at least 400 bp of homology to each of the gene flanking regions. For C-terminal tagging with fluorophores, cells were transformed with fluorophore tagging plasmids carrying at least 400 bp of homology to the C-terminal part of the gene and 3′UTR (Table S2). For expression of fluorescently tagged Ltc1, the full-length Ltc1 gene sequence was cloned using the Infusion cloning method into pAV714 (expression under *p^act1^* promoter) or pAV747 (expression under *p^pom1^* promoter; Vjestica et al., 2019). The Ltc1 truncated versions lacked the following amino acids: (1) construct missing GRAM domain Δ197-264aa, (2) construct missing StART domain Δ432-598aa, and (3) construct missing TM domain Δ646-764aa. All plasmids constructed in this study are listed in Table S2.

### Microscopy

Microscopy was performed by confocal spinning disc microscopy or widefield microscopy. The majority of imaging (unless otherwise stated) was performed using a spinning-disc microscope: DMI4000B inverted microscope equipped with an HCX PL APO 6100/1.46 NA oil objective and PerkinElmer Confocal system (including a Yokagawa CSU22 real-time confocal scanning head and solid-state laser lines) equipped with a cooled 14-bit frame transfer EMCCP C9100-50 camera. Stacks of z-series confocal sections were acquired at 0.4-µm intervals using the Volocity software. Unless otherwise indicated, images shown are single-plane views. All microscopy was performed at room temperature (∼23°C) unless otherwise indicated.

Widefield microscopy (Fig. 1 D) was performed on a DeltaVision platform (Applied Precision) composed of a customized Olympus IX-71 inverted microscope, an uPlan Apo 100×/1.4 NA oil objective, a 4.2Mpx PrimeBSI sCMOS camera (Photometrics), and an Insight SSI 7 color combined unit illuminator. Unless otherwise indicated, images shown are single-plane views.

Filipin and Calcofluor imaging (Fig. 1 G, Fig. 2 D, and Fig. 6 B) was done by widefield microscopy acquired with the Leica AS AF software (Leica MicroSystems).

Imaging of cells, unless otherwise stated, was performed on EMM-ALU pads solidified with 2% agarose.

Imaging of *sec8* mutant and FM4-64 uptake experiments was done in glass chambers coated with soybean lectin (*Glycine max* lectin, Sigma-Aldrich). Shortly chambers of 96-well plates (MGB096-1-2LG-L, Matrical Bioscience) were covered with a 100 µl of filtered lectin solution (100 µg/ml in water) and kept at room temperature for 4 h. Afterward, lectin was removed, and plates were washed three times with 200 µl of water and dried. To attach cells to the bottom of glass chamber, 100 µl of cells OD 0.2 was added to plate wells and incubated for 30 min. Afterward, excess of cells was removed and wells were washed two times with medium before microscopy.

### Fluorescence image quantification

Quantification of the PM:cytosol mCherry-D4H ratio was done on single-plane images. Briefly, a segmented line was drawn along the PM, and the cytoplasm area was marked using a 4-pixel-wide polygon selection tool. Mean fluorescence value was calculated for each selection and corrected for background fluorescence intensity. For quantification of tip:side ratio in mCherry-D4H–expressing cells, cell tips or cell sides were marked using a four-pixel-wide segmented line. Mean fluorescence value was calculated for each selection and corrected for background fluorescence intensity. Quantification of Ltc1-sfGFP expression was done on sum projection images of three medial z-sections. The cell borders were outlined, and mean fluorescence intensity was measured and corrected for background fluorescence. Quantification of Ltc1 puncta intensity was done on single focal plane images. Briefly, puncta area was marked, and mean fluorescence activity was measured for each selection and corrected for background fluorescence intensity.

For cell length and width measurements on calcofluor-stained cells, a line was drawn manually across the length and width of septated cells from the middle of one tip to the other, and the length measured using the Fiji Measure tool. Figures were prepared with Fiji (Schindelin et al., 2012), Plots of Data (Postma and Goedhart, 2019), and Adobe Illustrator.

### CLEM and tomography

CLEM was performed essentially as described in Kukulski et al. (2012). Briefly, 5 ml of cells grown in EMM-ALU were concentrated in 500 µl, and 2.5 µl of a 100 mM CK-666 stock in DMSO was added for 45 min to 1 h. The cells were further concentrated to a thick slurry and pipetted onto a 3-mm-wide, 0.1-mm-deep specimen carrier (Wohlwend type A) closed with a flat lid (Wohlwend type B) for high-pressure freezing with a Wohlwend HPF Compact 02. The carrier sandwich was disassembled in liquid nitrogen before freeze substitution. High-pressure frozen samples were processed by freeze substitution and embedding in Lowicryl HM20 using the Leica AFS 2 robot as described (Kukulski et al., 2012). 300-nm sections were cut with a diamond knife using a Leica Ultracut E microtome, collected in H_2_0, and picked up on carbon-coated 200 mesh copper grids (AGS160; Agar Scientific). For light microscopy, the grid was inverted onto a 1× PBS drop on a microscope coverslip, which was mounted onto a microscope slide and imaged on a DeltaVision platform (Applied Precision) composed of a customized inverted microscope (IX-71; Olympus), a uPlan Apochromat 100×/1.4 NA oil objective, a 4.2Mpx PrimeBSI sCMOS camera, and a color combined unit illuminator (Insight SSI 7; Social Science Insights). Images were acquired using softWoRx v.7.0 software (Applied Precision). The grid was then recovered, rinsed in H_2_O, and dried before post-staining with Reynolds lead citrate for 10 min. 15-nm protein A–coupled gold beads were adsorbed to the top of the section as fiducials for tomography. Transmission electron micrographs (TEM) were acquired on a FEI Tecnai 12 at 120 kV using a bottom mount FEI Eagle camera (4kx4k) at 6,800× or 9,300× magnification for correlation with the light microscopy image. For correlation, the peripheral D4H signal was used as guide to draw the cell PM on the light microscopy image, which was used to define the image magnification and rotation required to match it to the PM on corresponding TEM images. For tomographic reconstruction of regions of interest, tilt series were acquired at 18,500× magnification over a 60° to −60° tilt range where possible at 1° increments using the Serial EM software. For tomogram reconstruction, we used the IMOD software package with gold fiducial alignment (Mastronarde and Held, 2017).

### Colony forming unit assay

Colony forming unit assays were performed in order to assess the viability of cells treated with AmB. Cells were cultured to OD 0.4–0.6, pelleted, and resuspended to the final OD of 4 in YE5S medium containing either DMSO (1%) or CK-666 (500 μM). After 1 h of cultivation (25°C, shaking), AmB was added to the final concentration of 5 µg/ml, and the samples were further incubated for another hour. Afterward, cells were collected by centrifugation, washed two times with YE5S medium, and diluted to final OD of 0.00025. 100 µl of cells was plated on YE5S plates, and colonies were counted after 3 d of incubation at 30°C.

### Flow cytometry

Flow cytometry was performed on a BD Biosciences Fortessa analyzer. To stain for the dead cells in the population, cells were diluted in EMM-ALU to final a OD of 0.1, and 100 µl of cell suspension was mixed with 900 μl EMM-ALU containing 1 µg/ml propidium iodide. After 30 s of incubation, cells were analyzed by flow cytometry without gating during acquisition with 10,000 cells recorded for each sample. Data were analyzed using FlowJo software.

### Spotting assay

Sensitivity to AmB was assessed by spotting 5 µl of yeast culture at an OD of 0.25 and its fivefold serial dilutions on plates containing EMM-ALU with indicated AmB concentration. Growth was assessed after 72 h at 30°C.

### Phylogenetic analysis

Full-length sequences of all OSBP domain-containing proteins identified in the *S. pombe* and *S. cerevisiae* genome were aligned using Muscle (https://www.ebi.ac.uk/Tools/msa/muscle/), and a neighbor-joining tree without distance correction was derived.

### Estimation of the rates of sterol flow

The following considerations were made to obtain an estimate of the number of sterol molecules internalized: total PM area, 2*πr(l − *2*r*) + 4*πr*^2^ ≅ 125 µm^2^ for a 10-µm-long, 4-µm-wide *S. pombe* cell; total endosome surface, 50 × 4*πr*^2^ ≅ 2.5 µm^2^ for 50 endosomes of diameter 127 nm; estimated surface of a single sterol molecule, 0.25 nm^2^ (Scott, 2002); minimal PM sterol concentration when labeled by D4 and D4H, 30 mol%; number of sterol molecules per surface area at 30 mol%, 150 per 240 nm^2^ (Hofsäss et al., 2003); maximal PM sterol concentration when not labeled by D4H, 20 mol%; time of internalization, 1 h (3,600 s); and number of Ltc1 molecules in the cell, 5,000 (Carpy et al., 2014).

If the PM contains 30 mol% sterols in the inner leaflet, this represents ∼7.8 × 10^7^ sterol molecules per cell. A 10 mol% drop upon Arp2/3 inhibition thus represents ∼2.6 × 10^7^ sterol molecules. 50 mol% sterol on endosome represents ∼2.6 × 10^6^ molecules, i.e., 10% of the sterols depleted from the PM. As it takes ∼1h to deplete the PM from the D4H signal, this amounts to a rate of ∼7,250 molecules/s, or ∼1.4 sterol molecule per Ltc1 protein per s.

### Statistical analysis

Statistical analysis was performed using the Excel package. Statistical significance was determined using a two-sided *t* test. Data distribution was assumed to be normal, but this was not formally tested.

### Online supplemental material

Fig. S1 shows controls for lack of D4H toxicity, ketoconazole and terbinafine treatment, and microtubule depolymerization. Fig. S2 shows the list of deletion strains tested for their ability to relocate mCherry-D4H upon CK-666 treatment. Fig. S3 shows the list of YFP-tagged transmembrane proteins evaluated for colocalization with mCherry-D4H positive structures. Fig. S4 shows colocalization analysis of internal D4H signal with components of endo-membranes. Fig. S5 shows colocalization of GFP-Syb1 with internalized FM4-64, normal D4H distribution in *osh2Δ osh3Δ* double mutants, and relative expression levels of Ltc1-sfGFP under native, *p^pom1^*, and *p^act1^* promoters. Table S1 provides the list of strains used in this study. Table S2 provides the list of plasmids used in this study. Video 1, Video 2, and Video 3 show mCherry-D4H distribution during vegetative growth, upon CK-666 treatment, and upon CK-666 wash-out, respectively. Video 4 and Video 5 show colocalization of mCherry-D4H with Sec72-GFP and GFP-Syb1 during treatment with CK-666, respectively. Video 6 and Video 7 show mCherry-D4H distribution during vegetative growth and upon CK-666 treatment in *ltc1Δ* mutant, respectively.

## Supplementary Material

Table S1 provides the list of strains used in this study.

Table S2 provides the list of plasmids used in this study.
