# Supplementary material for: Sterol biosensor reveals LAM-family Ltc1-dependent sterol flow to endosomes upon Arp2/3 inhibition
Source: J Cell Biol. 2020 Apr 22;219(6):e202001147. doi: 10.1083/jcb.202001147 (PMC7265315; doi:10.1083/jcb.202001147)
Supplement: Table S1 — provides the list of strains used in this study. [file JCB_202001147_TableS1.docx]

**Supplementary Table S1**

| Strain | Genotype | Source |
| --- | --- | --- |
| YSM3440 | *h90 ura4+::p^act1^-mCherry-D4H* | This study |
| YSM3441 | *h90 ura4+::p^act1^-mCherry-D4* | This study |
| YSM3442 | *h+ ade6+::p^act1^-mCherry-D4H ura4+::p^act1^-sfGFP-RitC* | This study |
| YSM3443 | *h? leu1+::p^bip1^-GFP-AHDL ura4+::p^act1^mCherry-D4H* | This study |
| YSM3444 | *h+ ade6+::p^act1^-mCherry-D4H p^nmt1^-GFP-Tna1::KanMX6* | This study |
| YSM3445 | *h90 leu1+::p^nmt41^-rng2-CHD-GFP ade6+::p^act1^-mCherry-D4H* | This study |
| YSM3446 | *h+ ura4+::p^act1^-mCherry-D4H* | This study |
| YSM3447 | *h90 WT ura4-294* | Lab strain |
| YSM3448 | *h90 ltc1∆::HphMX6* | This study |
| YSM3449 | *h- arp2-1 ura4+::p^act1^-mCherry-D4H ade6-* | This study |
| YSM3450 | *h- end4Δ::KanMX4 ura4+::p^act1^-mCherry-D4H* | This study |
| YSM3451 | *h? end4::∆^tld^ NatMX6 pan1Δ^apw^ NatMX6 ura4+::p^act1^-mCherry-D4H* | This study |
| YSM3452 | *h- for3Δ::NatMX ura4+::p^act1^-mCherry-D4H* | This study |
| YSM3453 | *h- pil1Δ::NatMX ura4+::p^act1^-mCherry-D4H* | This study |
| YSM3454 | *h- scs2Δ::HphMX6 scs22Δ::NatMX6 ura4+::p^act1^-mCherry-D4H* | This study |
| YSM3455 | *h? wsp1∆::KanMX6 ura4+::p^act1^-mCherry-D4H* | This study |
| YSM3456 | *h90 fim1∆::KanMX6 ura4+::p^act1^-mCherry-D4H* | This study |
| YSM3457 | *h- Sec72-GFP::ura4+ ura4+::p^act1^-mCherry-D4H* | This study |
| YSM3458 | *h+ KanMX-p^Syb1^-GFP-Syb1 ade6+::p^act1^-mCherry-D4H* | This study |
| YSM3459 | *h- Anp1-GFP::ura4+ ura4+::p^act1^-mCherry-D4H* | This study |
| YSM3460 | *h- bch1Δ::KanMX4 cfr1Δ::BleMX6 ura4+::p^act1^-mCherry-D4H* | This study |
| YSM3461 | *h- apm1Δ::KanMX4 ura4+::p^act1^-mCherry-D4H* | This study |
| YSM3462 | *h? apm1Δ::KanMX4 bch1Δ::KanMX4 cfr1Δ::BleMX6 ura4+:: p^act1^-mCherry-D4H* | This study |
| YSM3463 | *h+ oss1Δ::KanMX4 ura4+::p^act1^-mCherry-D4H ade6- leu1-32* | This study |
| YSM3464 | *h+ osh2Δ::KanMX4 ura4+::p^act1^-mCherry-D4H ade6- leu1-32* | This study |
| YSM3465 | *h+ osh3Δ::KanMX4 ura4+::p^act1^-mCherry-D4H ade6- leu1-32* | This study |
| YSM3466 | *h+ osh41Δ::KanMX4 ura4+::p^act1^-mCherry-D4H ade6- leu1-32* | This study |
| YSM3467 | *h+ osh42Δ::KanMX4 ura4+::p^act1^-mCherry-D4H ade6- leu1-32* | This study |
| YSM3468 | *h90 osh6Δ::KanMX6 ura4+::p^act1^-mCherry-D4H* | This study |
| YSM3469 | *h- ltc2Δ::BleMX6 ura4+::p^act1^-mCherry-D4H* | This study |
| YSM3470 | *h+ ltc1Δ::HphMX6 ura4+::p^act1^-mCherry-D4H* | This study |
| YSM3471 | *h- leu1+::p^Bip1^-mCherry-AHDL Ltc1-sfGFP::KanMX6* | This study |
| YSM3472 | *h90 scs2Δ::KanMX6 scs22::HphMX6 leu1+::p^Bip1^-mCherry-AHDL Ltc1-sfGFP::KanMX6* | This study |
| YSM3473 | *h+ ltc1Δ::HphMX6 ura4+::p^Pom1^-Ltc1-sfGFP* | This study |
| YSM3474 | *h+ ltc1Δ::HphMX6 ura4+::p^pom1^-Ltc1GRAMΔ-sfGFP ade6+::p^act1^-mCherry-D4H* | This study |
| YSM3475 | *h+ ltc1Δ::HphMX6 ura4+::p^pom1^-Ltc1STARTΔ-sfGFP ade6+::p^act1^-mCherry-D4H* | This study |
| YSM3476 | *h+ ltc1Δ::HphMX6 ura4+::p^pom1^-Ltc1TMΔ-sfGFP ade6+::p^act1^-mCherry-D4H* | This study |
| YSM3477 | *h+ ltc1Δ::HphMX6 ura4+::p^act1^-Ltc1-sfGFP ade6+::p^act1^-mCherry-D4H* | This study |
| YSM3478 | *h+ ltc1Δ::HphMX6 ura4+::p^act1^-Ltc1GRAMΔ-sfGFP ade6+::p^act1^-mCherry-D4H* | This study |
| YSM3479 | *h+ ltc1Δ::HphMX6 ura4+::p^act1^-Ltc1STARTΔ-sfGFP ade6+::p^act1^-mCherry-D4H* | This study |
| YSM3480 | *h+ ltc1Δ::HphMX6 ura4+::p^act1^-Ltc1TMΔ-sfGFP ade6+::p^act1^-mCherry-D4H* | This study |
| YSM3481 | *h+ ltc1Δ ::HphMX6 p^nmt1^-GFP-Tna1::KanMX6 ura4+::p^act1^-mCherry-D4H* | This study |
| YSM3482 | *h+ ltc1Δ::HphMX6* | This study |
| YSM3483 | *h+ ltc2Δ::HphMX6* | This study |
| YSM3484 | *h+ ltc1Δ::HphMX6 ltc2Δ::HphMX6* | This study |
| YSM1371 | *h+ WT* | Lab strain |
| YSM3485 | *h+ ltc1Δ::HphMX6 ura4+::p^pom1^-Ltc1* | This study |
| YSM3486 | *h+ ltc1Δ::HphMX6 ura4+::p^pom1^-Ltc1^GRAMΔ^* | This study |
| YSM3487 | *h+ ltc1Δ::HphMX6 ura4+::p^pom1^-Ltc1^STARTΔ^* | This study |
| YSM3488 | *h+ ltc1Δ::HphMX6 ura4+::p^pom1^-Ltc1^TMΔ^* | This study |
| YSM3489 | *h- ltc1Δ::HphMX6 ura4+::p^act1^-Ltc1* | This study |
| YSM3490 | *h+ ltc1Δ::HphMX6 ura4+::p^act1^-Ltc1^GRAMΔ^* | This study |
| YSM3491 | *h- ltc1Δ::HphMX6 ura4+::p^act1^-Ltc1^STARTΔ^* | This study |
| YSM3492 | *h- ltc1Δ::HphMX6 ura4+::p^act1^-Ltc1^TMΔ^* | This study |
| YSM3493 | *h- ltc1Δ::HphMX6 ura4+::p^act1^-Ltc1* | This study |
| YSM3494 | *h- ltc1Δ::HphMX6 ura4+::p^act1^-Lam3* | This study |
| ­­YSM3495 | *h- ltc1Δ::HphMX6 ura4+::p^act1^-Lam2* | This study |
| YSM3496 | *h- ltc1Δ::HphMX6 ura4+::p^act1^-Lam4* | This study |
| YSM3497 | *h- ltc1Δ::HphMX6 ura4+::p^act1^-Lam1* | This study |
| YSM3498 | *h- ltc1Δ::HphMX6 ura4+::p^act1^-Lam5* | This study |
| YSM3499 | *h- ltc1Δ::HphMX6 ura4+::p^act1^-Lam6* | This study |
| YSM3500 | *h90 ade6+::p^psy1^-GFP-Psy1 ura4+::p^act1^-mCherry-D4H leu1-32* | This study |
| YSM3501 | *h90 ade6+::p^psy1^-GFP-Psy1 ura4+::p^act1^-mCherry-D4H ltc1Δ::HphMX6 leu1-32* | This study |
| YSM3503 | *h+ leu1+::p^bip1^-mCherry-AHDL ura4+::p^act1^-sfGFP-D4H ltc1Δ::HphMX6* | This study |
| YSM3504 | *h90 ura4+::p^act1^-mCherry-D4H Pil1-sfGFP::KanMX6* | This study |
| YSM3505 | *h90 ura4+::p^act1^-mCherry-D4H Pil1-sfGFP::KanMX6 ltc1Δ::HphMX6* | This study |
| YSM3506 | *h90 ura4+::p^act1^-mCherry-D4H leu1+::pSV40-GFP-Atb2* | This study |
| YSM3507 | *h- arf6Δ::KanMX6 ura4+: Pact1-mCherry-D4H ade6- leu1-32* | This study |
| YSM3508 | *h90 vps1Δ::KanMX6 ura4+::p^act1^-mCherry-D4H* | This study |
| YSM3509 | *h90 lsb5Δ::KanMX6 ura4+::p^act1^-mCherry-D4H* | This study |
| YSM3510 | *h90 hob1Δ::HphMX6 ura4+::p^act1^-mCherry-D4H* | This study |
| YSM3511 | *h90 hob3Δ::HphMX6 ura4+::p^act1^-mCherry-D4H* | This study |
| YSM3512 | *h+ sck1Δ::KanMX4 ura4+::p^act1^-mCherry-D4H ade6- leu1-32* | This study |
| YSM3513 | *h+ sck2Δ::KanMX4 ura4+::p^act1^-mCherry-D4H ade6- leu1-32* | This study |
| YSM3514 | *h- gad8Δ::HphMX6 ura4+::p^act1^-mCherry-D4H* | This study |
| YSM3515 | *h+ ppk33Δ::KanMX4 ura4+::p^act1^-mCherry-D4H ade6- leu1-32* | This study |
| YSM3516 | *h90 spk1Δ::KanMX6 ura4+::p^act1^-mCherry-D4H* | This study |
| YSM3517 | *h90 pmk1Δ::KanMX6 ura4+::p^act1^-mCherry-D4H* | This study |
| YSM3518 | *h+ sty1Δ::KanMX6 ade6+::p^act1^-mCherry-D4H ura4-D18* | This study |
| YSM3519 | *h+ rho3Δ::KanMX4 ura4+::p^act1^-mCherry-D4H ade6- leu1-32* | This study |
| YSM3520 | *h+ rho5Δ::KanMX4 ura4+::p^act1^-mCherry-D4H ade6- leu1-32* | This study |
| YSM3521 | *h90 spn2Δ::KanMX6 ura4+::p^act1^-mCherry-D4H* | This study |
| YSM3522 | *h90 are1Δ::bleMX6 are2Δ::NatMX6 ura4+::p^act1^-mCherry-D4H* | This study |
| YSM3523 | *h+ pDUAL-p^nmt1^-His6-Flag-YFP-Git3-ura4+ ade6+::p^act1^-mCherry-D4H* | This study |
| YSM3524 | *h+ pDUAL-p^nmt1^-His6-Flag-YFP-Bsu1-ura4+ ade6+::p^act1^-mCherry-D4H* | This study |
| YSM3525 | *h+ pDUAL-p^nmt1^-His6-Flag-YFP-Ght2-ura4+ ade6+::p^act1^-mCherry-D4H* | This study |
| YSM3526 | *h+ pDUAL-p^nmt1^-His6-Flag-YFP-Caf5-ura4+ ade6+::p^act1^-mCherry-D4H* | This study |
| YSM3527 | *h+ pDUAL-p^nmt1^-His6-Flag-YFP-Bfr12-ura4+ ade6+::p^act1^-mCherry-D4H* | This study |
| YSM3528 | *h+ pDUAL-p^nmt1^-His6-Flag-YFP-Ght3-ura4+ ade6+::p^act1^-mCherry-D4H* | This study |
| YSM3529 | *h+ pDUAL-p^nmt1^-His6-Flag-YFP-Str3-ura4+ ade6+::p^act1^-mCherry-D4H* | This study |
| YSM3530 | *h+ pDUAL-p^nmt1^-His6-Flag-YFP-Liz1-ura4+ ade6+::p^act1^-mCherry-D4H* | This study |
| YSM3531 | *h+ pDUAL-p^nmt1^-His6-Flag-YFP-Pma1-ura4+ ade6+::p^act1^-mCherry-D4H* | This study |
| YSM3532 | *h+ pDUAL-p^nmt1^-His6-Flag-YFP-Mok12-ura4+ ade6+::p^act1^-mCherry-D4H* | This study |
| YSM3533 | *h+ pDUAL-p^nmt1^-His6-Flag-YFP-Pmd1-ura4+ ade6+::p^act1^-mCherry-D4H* | This study |
| YSM3534 | *h+ pDUAL-p^nmt1^-His6-Flag-YFP-Sod2-ura4+ ade6+::p^act1^-mCherry-D4H* | This study |
| YSM3535 | *h+ pDUAL-p^nmt1^-His6-Flag-YFP-Mug86-ura4+ ade6+::p^act1^-mCherry-D4H* | This study |
| YSM3536 | *h+ pDUAL-p^nmt1^-His6-Flag-YFP-Mug73-ura4+ ade6+::p^act1^-mCherry-D4H* | This study |
| YSM3537 | *h+ pDUAL-p^nmt1^-His6-Flag-YFP-Spbc36.01c-ura4+ ade6+::p^act1^-mCherry-D4H* | This study |
| YSM3538 | *h+ pDUAL-p^nmt1^-His6-Flag-YFP-Spac997.17-ura4+ ade6+::p^act1^-mCherry-D4H* | This study |
| YSM3539 | *h+ pDUAL-p^nmt1^-His6-Flag-YFP-Wsc1-ura4+ ade6+::p^act1^-mCherry-D4H* | This study |
| YSM3540 | *h+ pDUAL-p^nmt1^-His6-Flag-YFP-Trp663-ura4+ ade6+::p^act1^-mCherry-D4H* | This study |
| YSM3541 | *h+ pDUAL-p^nmt1^-His6-Flag-YFP-Spcc16a11.01-ura4+ ade6+:: p^act1^-mCherry-D4H* | This study |
| YSM3542 | *h+ pDUAL-p^nmt1^-His6-Flag-YFP-Spcc794.06-ura4+ ade6+::p^act1^-mCherry-D4H* | This study |
| YSM3543 | *h+ pDUAL-p^nmt1^-His6-Flag-YFP-Spcc576.17c-ura4+ ade6+::p^act1^-mCherry-D4H* | This study |
| YSM3544 | *h+ pDUAL-p^nmt1^-His6-Flag-YFP-Spbc947.06c-ura4+ ade6+::p^act1^-mCherry-D4H* | This study |
| YSM3545 | *h+ pDUAL-p^nmt1^-His6-Flag-YFP-Spbc887.17-ura4+ ade6+::p^act1^-mCherry-D4H* | This study |
| YSM3546 | *h+ pDUAL-p^nmt1^-His6-Flag-YFP-Spbc530.15c-ura4+ ade6+::p^act1^-mCherry-D4H* | This study |
| YSM3547 | *h90 ura4+:: p^act1^-GFP-2xPH(PLCδ) ade6+:: p^act1^-mCherry-D4H* | This study |
| YSM3548 | *h90 ura4+:: p^act1^-GFP-LactC2 ade6+::p^act1^-mCherry-D4H* | This study |
| YSM3549 | *h+ Sec24-GFP-ura4+ ura4+::p^act1^-mCherry-D4H* | This study |
| YSM3550 | *h- Apl5-sfGFP::KanMX6 ura4+::p^act1^-mCherry-D4H* | This study |
| YSM3551 | *h- Apl6-sfGFP::KanMX6 ura4+::p^act1^-mCherry-D4H* | This study |
| YSM3552 | *h- Vps8-sfGFP::KanMX6 ura4+::p^act1^-mCherry-D4H* | This study |
| YSM3553 | *h+ ade6+::P^Nmt41^-sfGFP-Ypt5 ura4+::p^act1^-mCherry-D4H* | This study |
| YSM3554 | *h90 End4-sfGFP::KanMX6 ura4+: p^act1^-mCherry-D4H* | This study |
| YSM3555 | *h90 Clc1-sfGFP::KanMX6 ura4+::p^act1^-mCherry-D4H* | This study |
| YSM3556 | *h? ade6+::P^nmt41^-GFP-Ypt3-hph ura4+::p^act1^-mCherry-D4H* | This study |
| YSM3557 | *h- Sec3-GFP::KanMX6 ura4+::p^act1^-mCherry-D4H ade6- leu1-32* | This study |
| YSM3558 | *h- Sec5-GFP::KanMX6 ura4+::p^act1^-mCherry-D4H ade6- leu1-32* | This study |
| YSM3559 | *h- Exo70-GFP::KanMX6 ura4+::p^act1^-mCherry-D4H ade6- leu1-32* | This study |
| YSM3560 | *h? bgs4::ura4+ leu1+::GFP-Bgs4 ura4+::p^act1^-mCherry-D4H* | This study |
| YSM3561 | *h- Pmp3-sfGFP::KanMX6 ura4+::p^act1^-mCherry-D4H* | This study |
| YSM3562 | *h? KanMX-GFP-Syb1 sec8+::P^nmt81^-sec8-ura4+ ura4+::p^act1^-mCherry-D4H* | This study |
| YSM3711 | *h? scs2Δ::HphMX6 scs22Δ::NatMX6 ura4+::p^act1^-mCherry-D4H leu1+::p^bip1^-GFP-AHDL* | This study |
| YSM3712 | *h+ yap18Δ::KanMX6 ura4+::p^act1^-mCherry-D4H* | This study |
| YSM3713 | *h+ syp1Δ::KanMX6 ura4+::p^act1^-mCherry-D4H* | This study |
| YSM3714 | *h+ hhp1Δ::KanMX6 ura4+::p^act1^-mCherry-D4H* | This study |
| YSM3715 | *h90 ura4+::p^act1^-sfGFP-D4H* | This study |
| YSM1627 | *h- KanMX6-GFP-Syb1 ade6- leu-* | Lab strain |
| YSM3716 | *h? osh2Δ::KanMX osh3Δ::KanMX ura4+::p^act1^-mCherry-D4H* | This study |
