# Supplementary material for: Sterol biosensor reveals LAM-family Ltc1-dependent sterol flow to endosomes upon Arp2/3 inhibition
Source: J Cell Biol. 2020 Apr 22;219(6):e202001147. doi: 10.1083/jcb.202001147 (PMC7265315; doi:10.1083/jcb.202001147)
Supplement: Table S2 — provides the list of plasmids used in this study. [file JCB_202001147_TableS2.docx]

**Supplementary Table S2 List of plasmids generated in this study**

| Strain | Plasmid name | Plasmid description |
| --- | --- | --- |
| pSM2056 | *pACT1-mCherry-D4H* | Expression of mCherry-D4H biosensor under the control of constitutive actin promoter. Plasmid integrates to ura locus. |
| psM2299 | *pACT1-mCherry-D4* | Expression of mCherry-D4 biosensor under the control of constitutive actin promoter. Plasmid integrates to ura locus. |
| pSM2244 | *pACT1-sfGFP-D4H* | Expression of sfGFP-D4 biosensor under the control of constitutive actin promoter. Plasmid integrates to ura locus. |
| pSM2304 | *pFA6a-BLE-pil1* | Plasmid used for deletion of *pil1* gene. Confers resistance to zeocin. |
| pSM2434 | *pFA6a-KAN-scs2* | Plasmid used for deletion of *scs2* gene. Confers resistance to kanamycin. |
| pSM2435 | *pFA6a-BLE-scs2* | Plasmid used for deletion of *scs2* gene. Confers resistance to zeocin. |
| pSM2099 | *pFA6a-NAT-scs22* | Plasmid used for deletion of *scs22* gene. Confers resistance to nourseothricin. |
| pSM2317 | *pFA6a-BLE-wsp1* | Plasmid used for deletion of *wsp1* gene. Confers resistance to zeocin. |
| pSM2318 | *pFA6a-BLE-fim1* | Plasmid used for deletion of *fim1* gene. Confers resistance to zeocin. |
| pSM2437 | *pFA6a-BLE-cfr1* | Plasmid used for deletion of *cfr1* gene. Confers resistance to zeocin. |
| pSM2176 | *pFA6a-HPH-ltc1* | Plasmid used for deletion of *ltc1* gene. Confers resistance to hygromycin. |
| pSM2175 | *pFA6a-BLE-ltc2* | Plasmid used for deletion of *ltc2* gene. Confers resistance to zeocin. |
| pSM2313 | *pFA6a-BLE-vps1* | Plasmid used for deletion of *vps1* gene. Confers resistance to zeocin. |
| pSM2329 | *pFA6a-HPH-hob1* | Plasmid used for deletion of *hob1* gene. Confers resistance to hygromycin. |
| pSM2330 | *pFA6a-HPH-hob3* | Plasmid used for deletion of *hob3* gene. Confers resistance to hygromycin. |
| pSM2161 | *pFA6a-HPH-gad8* | Plasmid used for deletion of *gad8* gene. Confers resistance to hygromycin. |
| pSM2276 | *pFA6a-HPH-spk1* | Plasmid used for deletion of *spk1* gene. Confers resistance to hygromycin. |
| pSM2277 | *pFA6a-HPH-pmk1* | Plasmid used for deletion of *pmk1* gene. Confers resistance to hygromycin. |
| pSM2309 | *pFA6a-BLE-are1* | Plasmid used for deletion of *pmk1* gene. Confers resistance to zeocin. |
| pSM2310 | *pFA6a-NAT-are2* | Plasmid used for deletion of *are2* gene. Confers resistance to nourseothricin. |
| pSM2398 | *pACT1-Ltc1-sfGFP* | Plasmid used for expression of Ltc1-sfGFP under the control of strong actin promoter. Plasmid integrates to ura locus. |
| pSM2399 | *pACT1-Ltc1-sfGFP-GRAM* | Plasmid used for expression of Ltc1-sfGFP mutant lacking GRAM domain under the control of strong actin promoter. Plasmid integrates to ura locus. |
| pSM2400 | *pACT1-Ltc1-sfGFP-StART* | Plasmid used for expression of Ltc1-sfGFP mutant lacking StART-like domain under the control of strong actin promoter. Plasmid integrates to ura locus. |
| pSM2401 | *pACT1-Ltc1-sfGFP-TM* | Plasmid used for expression of Ltc1-sfGFP mutant lacking TM domain under the control of strong actin promoter. Plasmid integrates to ura locus. |
| pSM2420 | *pPOM1-Ltc1-sfGFP* | Plasmid used for expression of Ltc1-sfGFP under the control of weak pom1 promoter. Plasmid integrates to ura locus. |
| pSM2421 | *pPOM1-Ltc1-sfGFP-GRAM* | Plasmid used for expression of Ltc1-sfGFP mutant lacking GRAM domain under the control of weak pom1 promoter. Plasmid integrates to ura locus. |
| pSM2422 | *pPOM1-Ltc1-sfGFP-StART* | Plasmid used for expression of Ltc1-sfGFP mutant lacking StART-like domain under the control of weak pom1 promoter. Plasmid integrates to ura locus. |
| pSM2423 | *pPOM1-Ltc1-sfGFP-TM* | Plasmid used for expression of Ltc1-sfGFP mutant lacking TM domain under the control of weak pom1 promoter. Plasmid integrates to ura locus. |
| pSM2372 | *pACT1-Ltc1* | Plasmid used for expression of Ltc1 under the control of strong actin promoter. Plasmid integrates to ura locus. |
| pSM2373 | *pACT1-Ltc1-GRAM* | Plasmid used for expression of Ltc1 mutant lacking GRAM domain under the control of strong actin promoter. Plasmid integrates to ura locus. |
| pSM2374 | *pACT1-Ltc1-StART* | Plasmid used for expression of Ltc1 mutant lacking StART-like domain under the control of strong actin promoter. Plasmid integrates to ura locus. |
| pSM2375 | *pACT1-Ltc1-TM* | Plasmid used for expression of Ltc1 mutant lacking TM domain under the control of strong actin promoter. Plasmid integrates to ura locus. |
| pSM2426 | *pPOM1-Ltc1* | Plasmid used for expression of Ltc1 under the control of weak pom1 promoter. Plasmid integrates to ura locus. |
| pSM2427 | *pPOM1-Ltc1-GRAM* | Plasmid used for expression of Ltc1 mutant lacking GRAM domain under the control of weak pom1 promoter. Plasmid integrates to ura locus. |
| pSM2428 | *pPOM1-Ltc1-StART* | Plasmid used for expression of Ltc1 mutant lacking StART-like domain under the control of weak pom1 promoter. Plasmid integrates to ura locus. |
| pSM2429 | *pPOM1-Ltc1-TM* | Plasmid used for expression of Ltc1 mutant lacking TM domain under the control of weak pom1 promoter. Plasmid integrates to ura locus. |
| pSM2159 | *pACT1-ScLAM3* | Plasmid used for expression of *LAM3* gene from *S. cerevisiae* under the control of strong actin promoter. Plasmid integrates to ura locus. |
| pSM2165 | *pACT1-ScLAM2* | Plasmid used for expression of *LAM2* gene from *S. cerevisiae* under the control of strong actin promoter. Plasmid integrates to ura locus. |
| pSM2166 | *pACT1-ScLAM4* | Plasmid used for expression of *LAM4* gene from *S. cerevisiae* under the control of strong actin promoter. |
| pSM2161 | *pACT1-ScLAM1* | Plasmid used for expression of *LAM1* gene from *S. cerevisiae* under the control of strong actin promoter. |
| pSM2168 | *pACT1-ScLAM5* | Plasmid used for expression of *LAM5* gene from *S. cerevisiae* under the control of strong actin promoter. |
| pSM2169 | *pACT1-ScLAM6* | Plasmid used for expression of *LAM6* gene from *S. cerevisiae* under the control of strong actin promoter. |
| pSM2311 | *Pil1-sfGFP-Ctag* | Plasmid used for genomic tagging of *pil1* gene with sfGFP at C terminus. Conferes resistance to kanamycin. |
| pSM2120 | *Ltc1-sfGFP-Ctag* | Plasmid used for genomic tagging of *ltc1* gene with sfGFP at C terminus. Conferes resistance to kanamycin. |
| pSM2211 | *Alp5-sfGFP-Ctag* | Plasmid used for genomic tagging of *alp5* gene with sfGFP at C terminus. Conferes resistance to kanamycin. |
| pSM2212 | *Alp6-sfGFP-Ctag* | Plasmid used for genomic tagging of *alp6* gene with sfGFP at C terminus. Conferes resistance to kanamycin. |
| pSM2204 | *Vps8-sfGFP-Ctag* | Plasmid used for genomic tagging of *vps8* gene with sfGFP at C terminus. Conferes resistance to kanamycin. |
| pSM2305 | *End4-sfGFP-Ctag* | Plasmid used for genomic tagging of *end4* gene with sfGFP at C terminus. Conferes resistance to kanamycin. |
| pSM2312 | *Clc1-sfGFP-Ctag* | Plasmid used for genomic tagging of *clc1* gene with sfGFP at C terminus. Conferes resistance to kanamycin. |
